# Randomized, Double-Blind, Crossover Study Comparing the Bioavailability of 4 Ashwagandha (*Withania somnifera* (L.) Dunal) Extracts in Healthy Adults Under Fasting Condition

**DOI:** 10.1016/j.curtheres.2025.100805

**Published:** 2025-07-10

**Authors:** Priyank Rathi, Se-Kwon Kim

**Affiliations:** 1 Synergen Bio Private Limited, Pune, Maharashtra, India; 2 College of Science & Technology, Hanyang University, ERICA Campus, Ansan, Republic of Korea

**Keywords:** Ashwagandha, Bioavailability, Bioequivalence, Pharmacokinetics, Withaferin A, *Withania somnifera*, Withanolide A

## Abstract

**Background:**

*Withania somnifera* (L.) Dunal, commonly known as ashwagandha, is a well-known plant in ayurvedic medicine, widely valued for its therapeutic potential. Although numerous clinical studies have explored its diverse health benefits, limited data are available on the pharmacokinetic (PK) properties and comparative bioavailability of its key bioactive constituents in humans.

**Objective:**

This study aimed to evaluate and compare the oral bioavailability of 4 commercially standardized ashwagandha extracts under fasting conditions in healthy adults.

**Methods:**

This randomized, double-blind, 4-treatment, 4-period, 4-sequence, single dose, 4-way crossover study was conducted in 16 healthy human volunteers. Participants received a single oral dose of 1 of 4 ashwagandha extracts, with varying compositions of 35% (*Withania somnifera* [WS]-35) or 10% (WS-10) withanolide glycosides, or 5% (WS-5) or 2.5% (WS-2.5) withanolides, each standardized to deliver 185 mg of total withanolides. Seventeen blood samples were collected over a 24-hour period after dose administration. Plasma concentrations of withanolide A, withanoside IV, withaferin A, and total withanolides were quantified, and PK parameters were calculated.

**Results:**

*Withania somnifera*-35 had significantly superior bioavailability compared with the other extracts. The AUC_0–t_ for total withanolides per gram of WS-35 was 118.28, 226.11, and 267.83 times better than WS-10, WS-5, and WS-2.5 respectively. *Withania somnifera*-35 exhibited a significantly higher C_max_, longer half-life, extended mean residence time, and lower systemic clearance, attributable to its higher withanolide glycoside content.

**Conclusions:**

These findings emphasize the critical role of withanolide glycosides in determining the PK performance of ashwagandha supplements. The enhanced bioavailability of WS-35 supports its preferential use in therapeutic applications and provides a strong rationale for further investigation into dose-response relationships and the long-term efficacy of standardized, high-bioavailability formulations. Clinical Trial Registry of India identifier: CTRI/2020/10/028397.

## Graphical abstract

**Figure:**
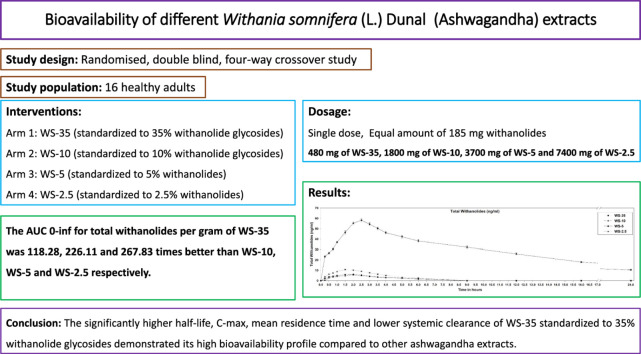

## Introduction

Ashwagandha (*Withania somnifera* (L.) Dunal [WS]), which belongs to the Solanaceae family, is a perennial shrub characterized by its broadly ovate to oblong leaves and long tuberous root. Ashwagandha is commonly found in warm and dry areas and is notable for the pharmacologically significant constituents found in its fruits, leaves, stem, and roots.^1^ Ashwagandha has been used in ayurveda and traditional Unani medicine for centuries as rasayana—a rejuvenator—which reportedly can promote longevity, vitality, and mental wellness.^2^ Charaka Samhita, a historical ayurvedic text, has detailed the historic utility of ashwagandha for improving vigor, cognitive function, and stress management.^3^ Among these pharmacologically important constituents, withanolides, comprising steroidal lactones and withanosides,^4^^,^^5^ are the primary active compounds contributing to the plant’s several pharmacologic activities.^6^ Primary withanolides (eg, withaferin A, withanolide A, and withanoside IV), along with sitoindosides and alkaloids were reportedly identified and isolated from WS. These bioactive compounds are predominantly concentrated in the mature leaves and roots of these plants. Studies reported approximately 62 major and minor primary and secondary metabolites from leaves and 48 major and minor primary and secondary metabolites from roots.^7^

The claims of traditional practices and texts on the medicinal properties of ashwagandha have been validated by modern pharmacologic studies. There is a significant demand of WS extracts for natural and ayurvedic medicine preparations, owing to their potential adaptogenic and immunomodulatory properties.^8^ Ashwagandha’s capability in influencing the hypothalamus-pituitary axis and brain neurotransmitters reportedly are effective in reducing stress, anxiety, improving mood and enhancing sleep quality.^9^ Furthermore, withanolides, the primary active component in WS, are known for their wide range of pharmacologic activities including anti-inflammatory, immunomodulating, anticancer, antidiabetic, antioxidant, aphrodisiac, antibacterial, and antineurodegenerative effects.^10^, ^11^, ^12^, ^13^, ^14^, ^15^ Although the usage of WS has been practiced for a long time, the WS extracts available in the market considerably differ in their active compound standardization, analytical methods, plant part being used, manufacturing process, and recommended dosage. These variabilities can potentially affect the absorption and therapeutic efficacy of WS extracts. Pharmacokinetic (PK) studies on the bioactive constituents of herbal drugs are important because they provide significant information regarding dosage, metabolism, and clinical use directions.^16^ Despite extensive clinical research and reporting of the improved clinical benefits of ashwagandha, limited PK data are available regarding the absorption and systematic availability of these key withanolides in humans, especially with respect to variability of extract formulations available in the commercial market, which necessitates further research into this aspect.

This human study assessed the oral bioavailability of active withanolides in blood plasma using 4 different commercially available WS extracts. The total withanolides content per dose was standardized to maintain consistent levels of withanolides across the different test products. This study aimed to elucidate the PK profile of withanolides from different WS extract formulations, which may improve the understanding of the relationship between these formulations and their bioavailability.

## Materials and Methods

### Study design

A randomized, double-blind, single-center clinical trial was carried out using a 4-period, 4-sequence, 4-treatment crossover model. Each participant received a single oral dose of 1 of the 4 standardized ashwagandha extract formulations during each study period with at least 7-day washout intervals between treatments. Healthy adult human subjects were enrolled in this study under fasting condition.

### Investigational products

This study evaluated 4 commercially available formulations of WS extract. The specifications of the investigational products used in this study were WS-35 (35% withanolide glycosides standardization), WS-10 (10% withanolide glycosides standardization), WS-5 (5% withanolides standardization), WS-2.5 (2.5% withanolides standardization). The investigational formulations were encapsulated in opaque green capsules of size “0.” Each capsule was designed to deliver a standardized dose of 185 mg of withanolides, as per the product specifications. To achieve this uniform dosage, the following quantities were used: WS-35 (480 mg), WS-10 (1800 mg), WS-5 (3700 mg), and WS-2.5 (7400 mg). All capsules had same color, appearance, and packaging. Blinding was made possible by equalizing the number of capsules using the prescribed dosage of investigational products and roasted rice powder. The selection of WS-35, WS-10, WS-5, and WS-2.5 was driven by the objective to evaluate how varying levels of withanolide standardization—ranging from high (WS-35), moderate (WS-10), low (WS-5), to very low (WS-2.5)—in WS (ashwagandha) extracts influence oral bioavailability and PK in humans, when administered at an equivalent dose of 185 mg total withanolides per treatment arm. The phytochemical composition of each extract is included in **Supplemental Table 1**.

Randomization was performed by an independent biostatistician using SAS (version 9.4, SAS Institute Inc., Cary, North Carolina, USA), ensuring a balanced design using block randomization based on the PROC PLAN procedure. The randomization schedule determined the order of group assignments during the 4 periods of the study. The pharmacy custodian dispensed the investigational products as per the randomization schedule in dispensing containers, prelabeled with “For clinical research use only” and including information on project number, period number, and subject number. Both the investigators, study personnel, and the subjects involved in assessments were blinded toward medications, thus minimizing potential bias in the study results.

### Ethical approval

Before the initiation of the study, the study protocol received ethical clearance from the Institutional Ethics Committee of Sai Sneh Hospital and Diagnostic Centre, Pune (DCGI Reg. No. ECR/989/Inst/MH/2017). The clinical study was carried out at Synergen Bio Private Limited, Pune, India, from November to December 2020 in accordance with the approved protocol (No. 006-20; approval date: September 29, 2020). This study was in compliance with the established clinical research standards. These included the International Council for Harmonisation of Technical Requirements for Pharmaceuticals for Human Use - Good clinical practice guidelines (2016), the Indian Council of Medical Research Ethical Guidelines for Biomedical Research on Human Subjects (2017), the Declaration of Helsinki (Fortaleza, Brazil, October 2013), G.S.R. 227(E) New Drugs and Clinical Trials Rules, 2019, and Central Drugs Standard Control Organization guidelines for Bioavailability and Bioequivalence Studies (March 2005). The trial was registered at Clinical Trial Registry of India (CTRI/2020/10/028397; registration date: October 13, 2020).

### Selection of participants for the study

After obtaining their informed consent, all volunteers were screened 21 days before dosing. A detailed verbal explanation covering the study’s objectives, procedures, investigational products, potential risks, participant responsibilities, and their rights were provided to all volunteers. Each volunteer was provided with chances to resolve any concerns regarding this study. The principal investigator received written consent from all willing volunteers before general screening and study-related procedures. Participants for this study were selected from the consented volunteers based on the following inclusion and exclusion criteria:

#### Inclusion criteria

Healthy adult male and/or female participants aged between 18 and 45 years underwent a screening procedure. The demographic characteristics of the participants were recorded. Participants were required to have a body mass index from 18.5 to 30.00 kg/m^2^ and a minimum weight of 50 kg. The participants were expected to be in good health as assessed by checking their personal medical history, vital signs, physical examination, normal 12-lead ECG, normal chest X-ray (posterior/anterior view). Urine screen for drug abuse (negative result) and an alcohol breath test were also considered. Effective communication skills, the ability to provide written informed consent, study protocol adherence, Identity proof, commitment to participate for the full study duration, and at least 14 hour fasting capacity were also necessary for inclusion in the study. The eligible participants based on the inclusion criteria were randomly assigned to the 4 formulations of WS extracts. Safety profiles were assessed during the screening phase and at specified time points as detailed in the protocol. Adverse events were documented through spontaneous report and open-ended questioning of participants at each visit. Clinical assessments included physical examination, vital signs, laboratory investigations, and ECGs, which were conducted as part of the clinical laboratory evaluation. The schedule of assessments is provided in **Supplemental Table 2**.

#### Exclusion criteria

Volunteers who had history of hypersensitivity to ashwagandha or related substances were predominantly excluded from the study. Those who were not able to comprehend the informed consent information were excluded from the study. Participants with a medical history or identified presence of significant medical conditions, such as cardiovascular, pulmonary, hepatic, renal, gastrointestinal (GI), endocrine, immunologic, dermatologic, neurologic, or psychiatric diseases, identified during the initial screening were also excluded. Participants with a history or presence of alcoholism, drug abuse, or any specific medical conditions, such as asthma, gastric or duodenal ulcers, thyroid disorders, adrenal dysfunction, organic intracranial lesions, or cancer, were also considered not eligible for inclusion in this study. Participants who had consumed substances such as tobacco, caffeine, or xanthine-containing foods within 48 hours before the study were also excluded.

### Sample size calculation

A power analysis was conducted using NCSS PASS (Kaysville, Utah, USA) to determine the minimum sample size needed to detect a statistically significant effect at a predefined significance level. This analysis considered factors such as power (set at 90%), significance level (set at 5%), and an expected effect size (0.6) based on previous studies. Based on this analysis, a minimum of 12 participants were found to be necessary to achieve sufficient statistical power. Considering a potential dropout rate of 20%, a total of 16 participants was considered an adequate sample size for this study.

### Study procedure

After a minimum overnight fasting period of 10.00 hours, participants were orally administered one of the following study formulations according to the randomization schedule for each study period: WS-35, WS-10, WS-5, or WS-2.5. Blood samples were collected before dose administration (0.00 hours) and at multiple time points after dosage (0.25, 0.5, 0.75, 1, 1.5, 2, 2.5, 3, 3.5, 4, 5, 6, 9, 12, 16, and 24 hours) to assess the plasma concentrations of ashwagandha extract. The collected plasma samples were stored at −80°C and analyzed for the active compounds using a validated HPLC-MS/MS protocol.

### Evaluation and statistics

As this was a single-dose study, PK analysis for each participant included the assessment of the area under the concentration–time curve (AUC_0–t_ and AUC_0–∞_), C_max_, and T_max_. Moreover, the elimination rate constant (k_e_), elimination half-life (t_1/2_), and additional PK parameters were also assessed. T_max_ values among different treatments were analyzed using the Kruskal-Wallis test and Dwass-Steel-Critchlow-Fligner analysis. Statistical analysis was performed in R version 4.1 (R Core Team, Vienna, Austria). A general linear model analysis for fixed factors was used for natural log-transformed PK variables to understand the significant variability in bioavailability among the studied individuals under different treatments. Statistical tests were conducted at 95% level of significance. Bioequivalence is generally assessed by comparing the population means of PK parameters such as AUC and C_max_. This approach, termed *mean bioequivalence*, involves computing a 90% confidence interval for the ratio of the geometric means of these parameters between the test and reference formulations.^17^ The mean ratios and corresponding 90% CIs were estimated for PK variables.

## Results

**Fig. 1:**
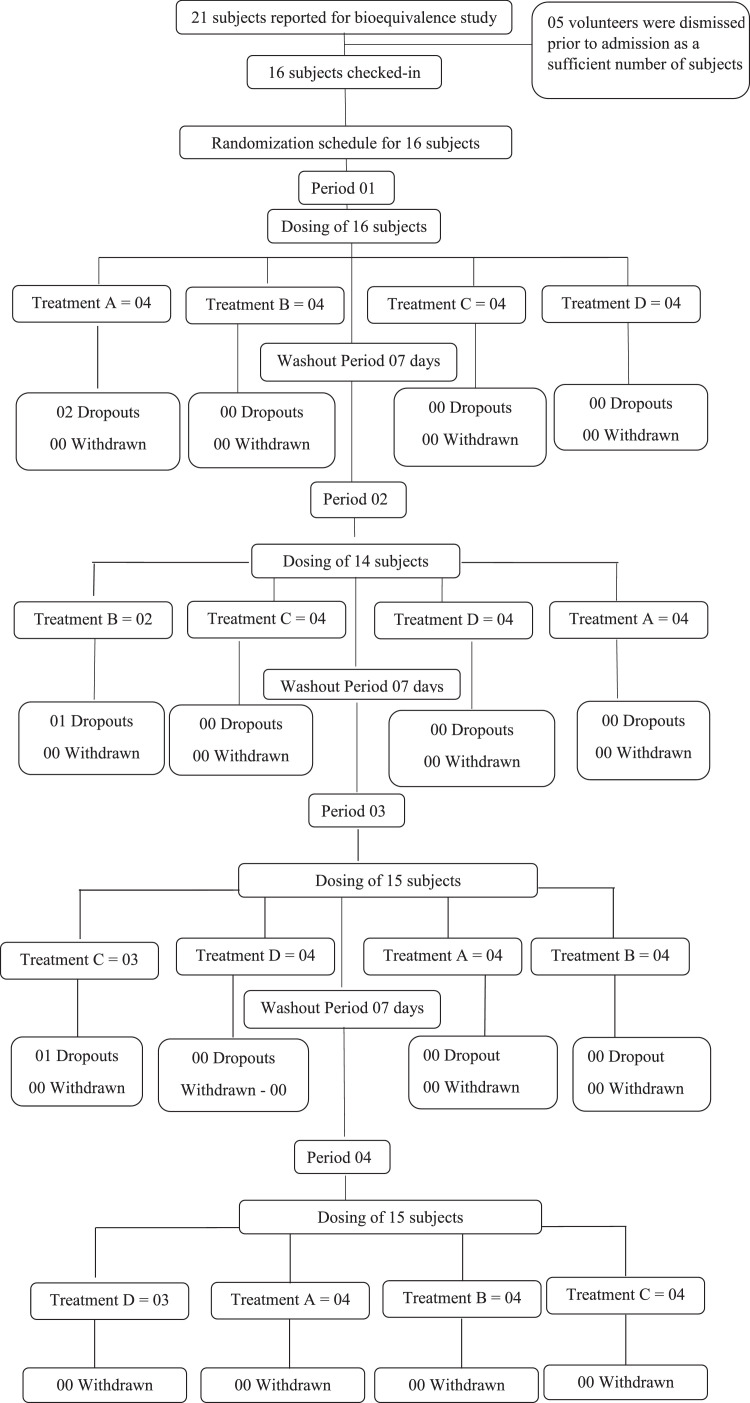
Disposition of participants flowchart.

Based on the inclusion and exclusion criteria, a total of 16 healthy participants were enrolled in the study. Randomization and group assignment is shown in Figure 1. General screening was carried out within the 21 days before dosing to select at least 16 healthy, adult, human subjects as per the protocol.

**Fig. 2:**
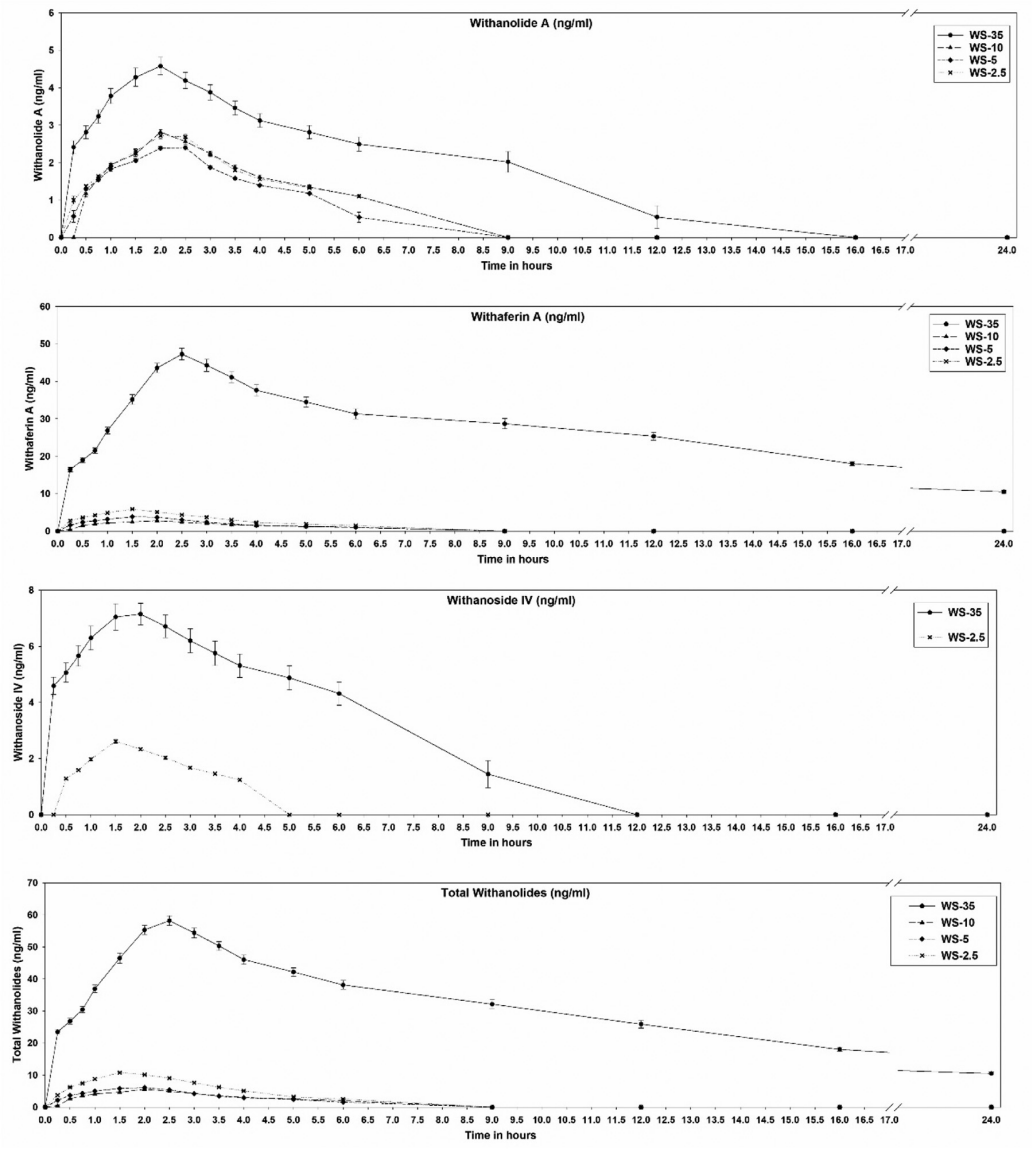
Plasma concentration–time curve of withanolide A, withaferin A, withanoside IV, and total withanolides after administration of 4 different formulations of *Withania somnifera* (WS) extracts.

**Fig. 3:**
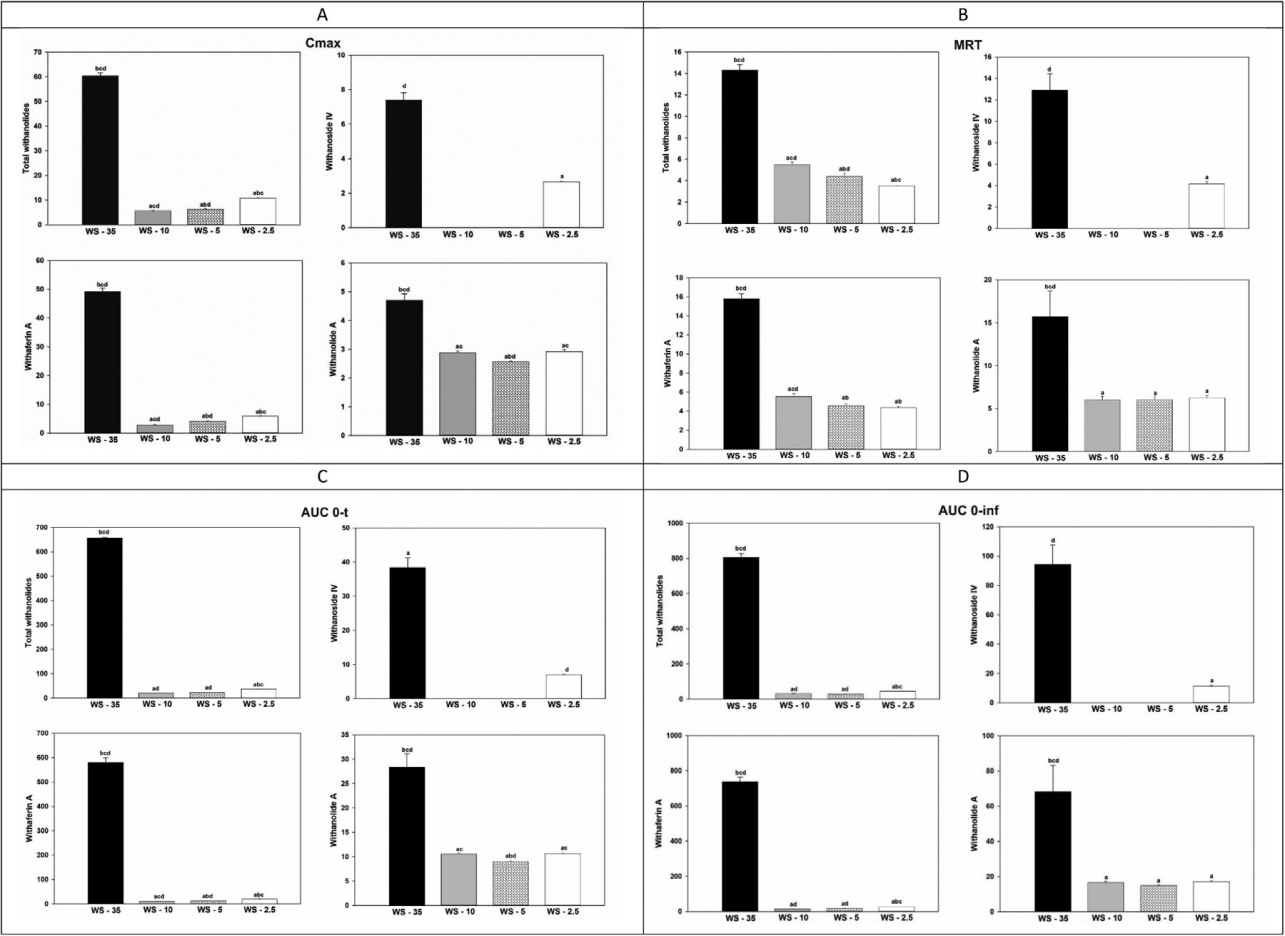
Representation of (A) C_max_, (B) mean residence time (MRT), (C) AUC_0–t_, and (D) AUC_0–∞_ of total withanolides, withanoside IV, withaferin A, and withanolide A after administration of 4 different *Withania somnifera* extracts (WS-35, WS-10, WS-5, and WS-2.5). Statistical significance compared with WS-35 represented by “a,” WS-10 by “b,” WS-5 by “c,” and WS-2.5 by “d.”.

Plasma concentrations of withanolide A, withaferin A, withanoside IV, and total withanolides were measured at various time points after a single oral administration of standardized doses (185 mg of withanolide glycosides) from 4 ashwagandha extract formulations (WS-35, WS-10, WS-5, and WS-2.5) in healthy individuals (Figure 2). Total withanolides were calculated by summing the concentrations of withanolide A, withaferin A, and withanoside IV. Pharmacokinetic parameters of these compounds in the blood plasma for each of the 4 extract formulations are represented in the Table 1. C_max_, mean residence time (MRT), AUC_0–t_, and AUC_0–∞_ of withanolide A, withaferin A, withanoside IV, and total withanolides of WS-35, WS-10, WS-5, and WS-2.5 are presented in Figure 3. Withanoside IV was not detected in WS-10 and WS-5. The mean AUC_0–t_ for total withanolides of 480 mg of WS-35 was 31.54, 29.33, and 17.37 times higher than those of 1800 mg for WS-10, 3700 mg of WS-5, and 7400 mg of WS-2.5, respectively. The mean AUC_0–∞_ for total withanolides of 480 mg of WS-35 was 26.04-, 27.49-, and 18-fold higher than those for 1800 mg of WS-10, 3700 mg of WS-5, and 7400 mg of WS-2.5, respectively. Comparing the AUC of total withanolides per 1 mg of WS-35, WS-10, WS-5, and WS- 2.5, mean AUC_0–t_ of WS-35 was 118.28, 226.11, and 267.83 times higher than those of WS-10, WS-5, and WS-2.5, respectively, and the mean AUC_0–∞_ of WS-35 was 97.64, 211.87, and 277.55 times higher than those of WS-10, WS-5, and WS-2.5, respectively.

**Table 1:** Comparison of pharmacokinetic parameters of withanolide A, withaferin A, withanoside IV, and total withanolides concentration in blood plasma with *Withania somnifera* extracts.

Parameter*	Product	Withanolide A	Withaferin A	Withanoside IV	Total withanolides
		Mean ± SEM	Mean ± SEM	Mean ± SEM	Mean ± SEM
Lambda z	WS-35	0.097 ± 0.015^b^^c^^d^	0.069 ± 0.003^b^^c^^d^	0.099 ± 0.012^d^	0.074 ± 0.003^b^^c^^d^
	WS-10	0.198 ± 0.014^a^	0.212 ± 0.017^a^	‡	0.215 ± 0.012^a^^d^
	WS-5	0.193 ± 0.012^a^	0.237 ± 0.013^a^	‡	0.272 ± 0.023^a^^d^
	WS-2.5	0.182 ± 0.011^a^	0.250 ± 0.016^a^	0.304 ± 0.015^a^	0.369 ± 0.005^a^^b^^c^
thalf	WS-35	10.761 ± 2.158^b^^c^^d^	10.244 ± 0.458^b^^c^^d^	8.810 ± 1.103^d^	9.644 ± 0.479^b^^c^^d^
	WS-10	3.759 ± 0.306^a^	3.539 ± 0.278^a^	‡	3.398 ± 0.247^a^^d^
	WS-5	3.832 ± 0.298^a^	3.062 ± 0.197^a^	‡	2.825 ± 0.245^a^^d^
	WS-2.5	4.029 ± 0.256^a^	2.929 ± 0.178^a^	2.407 ± 0.189^a^	1.884 ± 0.030^a^^b^^c^
Tmax†	WS-35	1.813 ± 0.063^b^^c^^d^	2.219 ± 0.102^c^^d^	1.750 ± 0.065^d^	2.219 ± 0.102^c^^d^
	WS-10	2.036 ± 0.036^a^	1.964 ± 0.036^c^^d^	‡	2.036 ± 0.036^c^^d^
	WS-5	2.200 ± 0.082^a^	1.700 ± 0.065^a^^b^^d^	‡	1.733 ± 0.067^a^^b^^d^
	WS-2.5	2.200 ± 0.082^a^	1.500 ± 0.000^a^^b^^c^	1.567 ± 0.067^a^	1.500 ± 0.000^a^^b^^c^
Cmax	WS-35	4.700 ± 0.225^b^^c^^d^	49.050 ± 1.318^b^^c^^d^	7.400 ± 0.423^d^	60.419 ± 1.172^b^^c^^d^
	WS-10	2.871 ± 0.071^a^^c^	2.786 ± 0.091^a^^c^^d^	‡	5.614 ± 0.147^a^^c^^d^
	WS-5	2.560 ± 0.032^a^^b^^d^	4.133 ± 0.142^a^^b^^d^	‡	6.340 ± 0.153^a^^b^^d^
	WS-2.5	2.927 ± 0.059^a^^c^	5.860 ± 0.087^a^^b^^c^	2.667 ± 0.040^a^	10.780 ± 0.128^a^^b^^c^
AUC0-t	WS-35	28.377 ± 2.731^b^^c^^d^	580.230 ± 18.368^b^^c^^d^	38.361 ± 2.938^a^	656.521 ± 17.852^b^^c^^d^
	WS-10	10.552 ± 0.220^a^^c^	10.336 ± 0.309^a^^c^^d^	‡	20.814 ± 0.478^a^^d^
	WS-5	8.998 ± 0.193^a^^b^^d^	13.201 ± 0.299^a^^b^^d^	‡	22.382 ± 0.346^a^^d^
	WS-2.5	10.663 ± 0.148^a^^c^	19.726 ± 0.434^a^^b^^c^	6.986 ± 0.116^d^	37.790 ± 0.513^a^^b^^c^
AUC0-inf	WS-35	68.143 ± 15.107^b^^c^^d^	737.755 ± 25.167^b^^c^^d^	94.309 ± 13.358^d^	804.968 ± 23.409^b^^c^^d^
	WS-10	16.570 ± 0.680^a^	15.433 ± 0.586^a^^d^	‡	30.917 ± 1.135^a^^d^
	WS-5	14.921 ± 0.549^a^	17.735 ± 0.439^a^^d^	‡	29.286 ± 1.177^a^^d^
	WS-2.5	17.113 ± 0.551^a^	25.828 ± 0.543^a^^b^^c^	11.306 ± 0.375^a^	44.712 ± 0.580^a^^b^^c^
AUCext	WS-35	39.766 ± 12.733^b^^c^^d^	157.526 ± 11.849^b^^c^^d^	55.948 ± 11.531^d^	148.447 ± 11.902^b^^c^^d^
	WS-10	6.019 ± 0.649^a^	5.097 ± 0.460^a^	‡	10.103 ± 0.907^a^^c^^d^
	WS-5	5.923 ± 0.523^a^	4.534 ± 0.350^a^	‡	6.905 ± 1.029^a^^b^
	WS-2.5	6.450 ± 0.484^a^	6.102 ± 0.386^a^	4.320 ± 0.378^a^	6.922 ± 0.157^a^^b^
MRT	WS-35	15.729 ± 2.994^b^^c^^d^	15.795 ± 0.515^b^^c^^d^	12.878 ± 1.548^d^	14.307 ± 0.532^b^^c^^d^
	WS-10	6.024 ± 0.382^a^	5.541 ± 0.305^a^^d^	‡	5.480 ± 0.277^a^^c^^d^
	WS-5	6.026 ± 0.348^a^	4.561 ± 0.224^a^	‡	4.395 ± 0.291^a^^b^^d^
	WS-2.5	6.240 ± 0.288^a^	4.356 ± 0.158^a^^b^	4.156 ± 0.240^a^	3.504 ± 0.027^a^^b^^c^
Vz	WS-35	42.628 ± 1.967^b^^c^^d^	3.736 ± 0.152^b^^c^^d^	27.110 ± 2.518^d^	3.212 ± 0.140^b^^c^^d^
	WS-10	59.804 ± 2.914^a^	60.688 ± 3.337^a^^c^^d^	‡	29.108 ± 1.268^a^^c^^d^
	WS-5	67.360 ± 2.587^a^	45.949 ± 2.487^a^^b^^d^	‡	25.176 ± 1.314^a^^b^^d^
	WS-2.5	62.190 ± 2.354^a^	30.302 ± 1.791^a^^b^^c^	56.169 ± 2.509^a^	11.278 ± 0.248^a^^b^^c^
Cl	WS-35	3.919 ± 0.483^b^^c^^d^	0.255 ± 0.009^b^^c^^d^	2.574 ± 0.323^d^	0.233 ± 0.007^b^^c^^d^
	WS-10	11.374 ± 0.397^a^	12.270 ± 0.585^a^^d^	‡	6.089 ± 0.224^a^^d^
	WS-5	12.616 ± 0.429^a^	10.523 ± 0.264^a^^d^	‡	6.463 ± 0.261^a^^d^
	WS-2.5	10.964 ± 0.343^a^	7.207 ± 0.153^a^^b^^c^	16.579 ± 0.470^a^	4.147 ± 0.054^a^^b^^c^
Ke	WS-35	0.097 ± 0.015^b^^c^^d^	0.069 ± 0.003^b^^c^^d^	0.099 ± 0.012^d^	0.074 ± 0.003^b^^c^^d^
	WS-10	0.198 ± 0.014^a^	0.212 ± 0.017^a^	‡	0.215 ± 0.012^a^^d^
	WS-5	0.193 ± 0.012^a^	0.237 ± 0.013^a^	‡	0.272 ± 0.023^a^^d^
	WS-2.5	0.182 ± 0.011^a^	0.250 ± 0.016^a^	0.304 ± 0.015^a^	0.369 ± 0.005^a^^b^^c^

a statistically significant at 95% level of significance when compared with WS-35.

b statistically significant at 95% level of significance when compared with WS-10.

c statistically significant at 95% level of significance when compared with WS-5.

d statistically significant at 95% level of significance when compared with WS-2.5.

⁎ General linear model analysis for fixed factors.

† Dwass-Steel-Critchlow-Fligner multiple comparison test.

‡ Withanoside IV was not detected for WS-10 and WS-5.

Among the tested formulations, WS-35 exhibited the highest AUC_0–t_ for total withanolides, which was significantly higher than those of WS-10, WS-5, and WS-2.5 (*P* < 0.001). AUC is a measurement of overall bioavailability of a drug representing the total quantity of active compound that enters the systemic circulation. This elevated AUC in WS-35 can be attributed to its higher peak C_max_ and an extended t_1/2_ compared with the other test products. C_max_ values for WS-35 were significantly higher (*P* < 0.001), with higher peak plasma concentration. The prolonged MRT and t_1/2_ for WS-35 (*P* < 0.001) illustrates its sustained systemic exposure. A more concentrated extract contains more free, unbound active compound exposed at the point of digestion and lead to better absorption in the gut.

Statistical analysis of the data was conducted using a generalized linear model with a fixed effects model applied to the log-transformed AUC and C_max_ values. Statistical analysis found that the AUC, C_max_, and T_max_ values were significantly different between treatments (*P* < 0.05), with post hoc pairwise comparison indicating WS-35 having a peak concentration that was reached rapidly and consistently. Bioequivalence was assessed by ensuring that the 90% CIs for both AUC and C_max_ of the test formulation fell within the accepted range of 80% to 125% relative to the reference formulation. **Supplemental Table 3** shows that 90% CI for AUC of products WS-10, WS-5, and WS-2.5 fell in this range, indicating that they were not bioequivalent to WS-35. The investigational ashwagandha extract formulations were found to be well tolerated after a single-dose administration in healthy, adult participants under fasting conditions. No severe, serious, or life-threatening adverse events were reported throughout the study duration.

## Discussion

Absorption after oral administration involves a series of complex processes, with both the rate and extent of absorption affected by numerous variables. These include physicochemical properties (pKa, solubility, stability, diffusivity, lipophilicity, surface area, particle size, and crystalline structure), physiologic conditions like GI pH, blood flow within GI tract, gastric emptying rate, intestinal transit time, and absorption mechanisms), and formulation-specific factors such as the dosage form (tablet, capsule, solution, suspension, emulsion, and gel).^18^^,^^19^

In this study, although each ashwagandha formulation was standardized to deliver 185 mg of total withanolides, significant variability in bioavailability was observed. Earlier studies have described the possibility for withanolide and withanolide glycoside interconversion and its metabolism.^20^ Detailed studies in this area will help to explain the differences in PK data seen between the test products and its analytes.

High solubility in physiologic conditions, along with a higher concentration of active drugs in intestinal fluid, provides a higher concentration gradient, which can drive the absorption of orally administered compounds.^21^ The higher withanolide glycoside content of WS-35 potentially contributed to its superior bioavailability. Different ratios of major withanolides in each extract, coupled with variable intestinal permeability of these compounds, may also affect their absorption.^18^^,^^22^^,^^23^ For example, an in vitro permeability study of ashwagandha withanolides in Madin-Darby canine kidney cells^24^ found that withanolide A, withanone, 1,2-deoxy-withastramonolide, and withanolide B were highly permeable. However, withanoside IV, withanoside V, and withaferin A had low permeability. Thus, the matrix and composition of WS-35 might have enhanced the dissolution and transport of active constituents across the intestinal walls.

Metabolic stability also plays a key role in systemic exposure. A PK study by Dai et al^20^ using withaferin A 5mg/kg administered intravenously and 10mg/kg administered orally over 24 hours in Sprague-Dawley rats reported rapid metabolism in rat and human liver microsomes (half-life 5.6 minutes). In spite of permeability in Caco-2 cells, the extensive first-pass metabolism of withaferin A may explain its limited oral bioavailability. Singh et al^25^ tested the bioavailability of withaferin A (purity 99%) in Sprague-Dawley rats, administering it orally at 25 mg/kg and intravenously at 2 mg/kg. They found oral bioavailability was low (about 5%) despite rapid systematic distribution after intravenous administration. The distribution of a compound within the body is largely influenced by its physicochemical characteristics and for some compounds, in part, by the contribution of transporter proteins.^26^ In a Phase I dose-escalation study conducted by Pires et al^27^ involving 13 participants, withaferin A was not detectable in plasma, even at the highest dose level tested. In contrast, our study successfully detected withaferin A in blood plasma across all ashwagandha extract formulations. One of the reasons for this difference could be attributed to the analytical methods—specifically, the HPLC method used by Pires et al,^27^ which had low level of specificity and sensitivity of 50 ng/mL, whereas our developed and validated LC-MS/MS method had an improved limit of quantification with lower limit of quantitation of 1.0 ng/mL for withanoside IV and withanolide A and 0.5 ng/mL for withaferin A in plasma.

To access bioequivalence, the mean value of PK markers such as AUC and C_max_ are typically compared across treatments using accepted statistical thresholds.^28^ For each component, the mean ratio and 90% CI are calculated. To establish bioequivalence, both AUC and C_max_ for the test product should be within 80% to 125% of the reference product using a 90% CI. In this study, the 90% CI for AUC ratios of WS-2.5, WS-5, and WS-10 with respect to WS-35 did not fall in the range 80% to 125%, indicating that WS-35 is not bioequivalent to WS-2.5, WS-5, and WS-10. The findings from this study advocate for stricter standardization practices and suggest that WS-35 may provide superior clinical efficiency, attributed to its favorable PK profile.

No control group was included in the study, as the primary objective was to compare the PK profiles and assess the relative bioavailability of 4 standardized WS extracts rather than to evaluate the therapeutic efficacy. Moreover, the control itself would not contain any active withanolides, and hence it will not provide meaningful data to compare with the studied formulations. A crossover design was included in this study instead, which provided direct within-subject comparison across formulations, which potentially reduced the interindividual variability, permitting a robust evaluation of formulation-dependent differences in systemic exposure.

### Strengths and limitations of the study

The plasma levels of 3 key withanolides, withaferin A, withanolide A, and withanoside IV, were quantified using a highly sensitive, validated LC-MS/MS analytical protocol developed specifically for this study. The method had high sensitivity with a lower limit of quantitation of 0.5 ng/mL for withaferin A and 1.0 ng/mL for both withanolide A and withanoside IV, enabling the accurate detection of plasma concentrations for all 4 WS extract formulations.^29^

Although equivalent dosage of total withanolides of 4 different ashwagandha extracts were administered, the wide variability in the PK parameters call for a uniform standard analysis method for product standardization. Although USP-35-NF 30^30^ is widely recognized as a standardized analytical method for WS, it only includes a limited set of marker compounds such as withanoside IV, physagulin D, 27-hydroxywithanone, withanoside V, withanoside VI, withaferin A, withastramonolide, withanolide A, withanone, and withanolide B. This narrow scope represents a significant limitation given the extensive diversity of phytochemicals identified in ashwagandha.

The study was limited to healthy male participants. Although this approach strengthens the internal comparison of formulations, it restricts generalizability. Future studies should consider a broader demographic group including female population to assess the sex-based differences in PK and bioavailability and also to increase generalizability.

Based on the USP method of analysis, WS-35, WS-10, WS-5, and WS-2.5 contain 7.5%, 0.18%, 0.29%, and 0.24% total withanolides, respectively, and the AUC_0–t_ and AUC_0–∞_ per milligram of ashwagandha extracts were 18.24 and 22.36 for WS-35, 6.42 and 9.54 for WS-10, 2.09 and 2.73 for WS-5, and 2.13 and 2.52 for WS-2.5, respectively. Moreover, standardization of products with uniform markers should be considered for future studies as different analysis methods were used in product specifications or label claims. Withanolides undergo extensive metabolism in vivo, and the secondary metabolites in plasma or withanolides in urine and feces were not measured in the study. Given that the study was performed only in men, there may be potential for a change of PK profile in women. Further studies in larger and multiracial populations with repeat dosage regimens are recommended.

## Conclusions

This study evidently reports the presence of key withanolides such as withanoside IV, withaferin A, and withanolide A in human plasma after oral intake of the 4 standardized WS extract formulations. It is evident from this analysis that the PK profiles varied significantly, despite the administration of equivalent total withanolide dose.

At a standardized dosage of withanolides, WS-35 had the highest systemic bioavailability, as evident from the significantly greater AUC, C_max_, and MRT. Also, the elimination half-life was longer in WS-5, WS-10, and WS-2.5 compared with WS-35. The results from this study illustrate the importance of extract composition in determining the efficiency of absorption and systemic exposure. It is also notable that withanoside IV was not detected in WS-10, WS-5, and WS-2.5, showing that certain extract types may lack key bioavailable components. Absence of bioequivalence in extracts such as WS-10, WS-5, and WS-2.5, compared with WS-35, illustrates the superiority of WS-35 in delivering consistent levels of bioactive plasma components. Among the formulations studied, it is clear that WS-35 had superior PK performance, illustrating that higher withanolide glycoside concentration leads to enhanced systemic exposure. Findings from this study support the use of standardized, highly potent extracts for strong and improved clinical effectiveness and hence provide a foundation for future dose optimization.

## Funding

This research received no specific grant from any funding agency in the public, commercial, or not-for-profit sectors.

## Author Contributions

Priyank Rathi conceived and designed the study, performed the investigation. Se-Kwon Kim: analyzed and interpreted the data, wrote the paper. All authors read and approved the final manuscript.

## Ethics Approval and Consent to Participate

The study was conducted in accordance with the Declaration of Helsinki after obtaining approval from Institutional Ethics Committee of Sai Sneh Hospital and Diagnostic Centre, Pune. Before any study-related screening procedures, written informed consent was obtained by the principal investigator from each patient before enrolling in the study.

## Declaration of competing interest

The authors declare that they have no known competing financial interests or personal relationships that could have appeared to influence the work reported in this paper.

## Appendix Supplementary materials

Image, application 1

Image, application 2

Image, application 3
