# Supplementary material for: Randomized, Double-Blind, Crossover Study Comparing the Bioavailability of 4 Ashwagandha (Withania somnifera (L.) Dunal) Extracts in Healthy Adults Under Fasting Condition
Source: Curr Ther Res Clin Exp. 2025 Jul 10;103:100805. doi: 10.1016/j.curtheres.2025.100805 (PMC12337022; doi:10.1016/j.curtheres.2025.100805)
Supplement: Supplementary file 1 [file mmc1.docx]

| Supplemental Table 1: Phyto-Chemical Analysis of Ashwagandha extracts as per Certificate of Analysis | | | | |
| --- | --- | --- | --- | --- |
| Ashwagandha extract | Parts used | Phyto-Chemical Analysis as per Certificate of Analysis | Result | Specification |
| WS-35 | Dried root and leaves | Total Withanolide Glycosides | 35.32% by HPLC | NLT 35% |
| WS-10 | Dried root and leaves | Withanolide Glycosides by HPLC-PDA | 12.94% | ≥ 10% (w/w) |
|  |  | Oligosaccharides by HPLC-PDA | 34.25% | ≥ 32% |
|  |  | Withanolide aglycone (as withaferin A) by HPLC-PDA | 0.35% | ≤ 0.5% (w/w) |
| WS-5 | Root | Total Withanolides (%w/w) by HPLC | 5.27% | ≥5% |
|  |  | Withaferin A by HPLC | Below Detection Levels (BDL) | <0.1% |
| WS-2.5 | Whole Herb | Withanolides by HPLC | 2.63% w/w | NLT 2.5% w/w |
