# Supplementary material for: Randomized, Double-Blind, Crossover Study Comparing the Bioavailability of 4 Ashwagandha (Withania somnifera (L.) Dunal) Extracts in Healthy Adults Under Fasting Condition
Source: Curr Ther Res Clin Exp. 2025 Jul 10;103:100805. doi: 10.1016/j.curtheres.2025.100805 (PMC12337022; doi:10.1016/j.curtheres.2025.100805)
Supplement: Supplementary file 2 [file mmc2.docx]

Supplemental Table 2. Schedule of Assessments

| **Activities** | **Screening** | **Period 01** | | | | **Period 02** | | | | **Period 03** | | | | **Period 04** | | |
| --- | --- | --- | --- | --- | --- | --- | --- | --- | --- | --- | --- | --- | --- | --- | --- | --- |
| **Day** | **D-20 to D01** | **D01** | **D02** | **D03** | **D08** | | **D09** | **D10** | **D15** | | **D16** | **D17** | **D22** | | **D23** | **D24** |
| Screening consent document | * | - | - | - | - | | - | - | - | | - | - | - | | - | - |
| Demographic data | * | - | - | - | - | | - | - | - | | - | - | - | | - | - |
| Blood sample for hematology, biochemistry and serological Examination | * | - | - | - | - | | - | - | - | | - | - | - | | - | - |
| Urine analysis | * | - | - | - | - | | - | - | - | | - | - | - | | - | - |
| ECG | * | - | - | - | - | | - | - | - | | - | - | - | | - | - |
| Clinical examination along with vital signs | * | * | - | * | * | | - | * | * | | - | * | * | | - | * |
| Vital signs | - | - | * | - | - | | * | - | - | | * | - | - | | * | - |
| Chest X ray (P/A view)^**^ | * | - | - | - | - | | - | - | - | | - | - | - | | - | - |
| Study informed consent document | - | * | - | - | - | | - | - | - | | - | - | - | | - | - |
| Review against inclusion/ exclusion criteria | - | * | - | - | - | | - | - | - | | - | - | - | | - | - |
| Urine sample for drugs of abuse | - | * | - | - | * | | - | - | * | | - | - | * | | - | - |
| Alcohol breath test | - | * | - | - | * | | - | - | * | | - | - | * | | - | - |
| Check-in | - | * | - | - | * | | - | - | * | | - | - | * | | - | - |
| Pre-dose sampling | - | - | * | - | - | | * | - | - | | * | - | - | | * | - |
| Dosing | - | - | * | - | - | | * | - | - | | * | - | - | | * | - |
| Post-dose sampling | - | - | * | * | * | | * | * | * | | * | * | * | | * | * |
| Medical event monitoring | * | * | - | - | - | | - | - | - | | - | - | - | | - | - |
| Adverse events monitoring | - | - | * | * | * | | * | * | * | | * | * | * | | * | * |
| Post-study safety sample | - | - | - | - | - | | - | - | - | | - | - | - | | - | * |
| Check-out | - | - | - | * | - | | - | * | - | | - | * | - | | - | * |
| *Activity done on this day. **Within 365days prior to period 01 dosing. | | | | | | | | | | | | | | | | |
