# Supplementary material for: Randomized, Double-Blind, Crossover Study Comparing the Bioavailability of 4 Ashwagandha (Withania somnifera (L.) Dunal) Extracts in Healthy Adults Under Fasting Condition
Source: Curr Ther Res Clin Exp. 2025 Jul 10;103:100805. doi: 10.1016/j.curtheres.2025.100805 (PMC12337022; doi:10.1016/j.curtheres.2025.100805)
Supplement: Supplementary file 3 [file mmc3.docx]

Supplemental Table 3. Statistical comparisons of pharmacokinetic parameters for Ashwagandha extracts

| **Pairwise Comparisons** | | | | | | | | |
| --- | --- | --- | --- | --- | --- | --- | --- | --- |
| Dependent Variable | | | | Mean Difference | Std. Error | Sig.^d^ | 90% Confidence Interval for Difference^d^ | |
|  |  |  |  |  |  |  | Lower Bound | Upper Bound |
| Lambda-z | Withanolide A | WS-35 | WS-10 | -.866^*^ | 0.116 | 0.000 | -1.058 | -0.674 |
|  |  |  | WS-5 | -.844^*^ | 0.114 | 0.000 | -1.033 | -0.656 |
|  |  |  | WS-2.5 | -.786^*^ | 0.114 | 0.000 | -0.974 | -0.597 |
|  |  | WS-10 | WS-35 | .866^*^ | 0.116 | 0.000 | 0.674 | 1.058 |
|  |  |  | WS-5 | 0.022 | 0.118 | 0.854 | -0.173 | 0.216 |
|  |  |  | WS-2.5 | 0.080 | 0.118 | 0.497 | -0.115 | 0.275 |
|  |  | WS-5 | WS-35 | .844^*^ | 0.114 | 0.000 | 0.656 | 1.033 |
|  |  |  | WS-10 | -0.022 | 0.118 | 0.854 | -0.216 | 0.173 |
|  |  |  | WS-2.5 | 0.059 | 0.116 | 0.614 | -0.133 | 0.250 |
|  |  | WS-2.5 | WS-35 | .786^*^ | 0.114 | 0.000 | 0.597 | 0.974 |
|  |  |  | WS-10 | -0.080 | 0.118 | 0.497 | -0.275 | 0.115 |
|  |  |  | WS-5 | -0.059 | 0.116 | 0.614 | -0.250 | 0.133 |
|  | Withaferin A | WS-35 | WS-10 | -1.089^*^ | 0.116 | 0.000 | -1.281 | -0.897 |
|  |  |  | WS-5 | -1.219^*^ | 0.114 | 0.000 | -1.407 | -1.031 |
|  |  |  | WS-2.5 | -1.265^*^ | 0.114 | 0.000 | -1.454 | -1.077 |
|  |  | WS-10 | WS-35 | 1.089^*^ | 0.116 | 0.000 | 0.897 | 1.281 |
|  |  |  | WS-5 | -0.130 | 0.118 | 0.273 | -0.324 | 0.065 |
|  |  |  | WS-2.5 | -0.176 | 0.118 | 0.137 | -0.371 | 0.019 |
|  |  | WS-5 | WS-35 | 1.219^*^ | 0.114 | 0.000 | 1.031 | 1.407 |
|  |  |  | WS-10 | 0.130 | 0.118 | 0.273 | -0.065 | 0.324 |
|  |  |  | WS-2.5 | -0.046 | 0.116 | 0.690 | -0.238 | 0.145 |
|  |  | WS-2.5 | WS-35 | 1.265^*^ | 0.114 | 0.000 | 1.077 | 1.454 |
|  |  |  | WS-10 | 0.176 | 0.118 | 0.137 | -0.019 | 0.371 |
|  |  |  | WS-5 | 0.046 | 0.116 | 0.690 | -0.145 | 0.238 |
|  | Withanoside IV | WS-35 | WS-10 | .^b^ |  |  |  |  |
|  |  |  | WS-5 | .^b^ |  |  |  |  |
|  |  |  | WS-2.5 | -1.212^*^ | 0.114 | 0.000 | -1.401 | -1.024 |
|  |  | WS-10 | WS-35 | .^c^ |  |  |  |  |
|  |  |  | WS-5 | .^b,c^ |  |  |  |  |
|  |  |  | WS-2.5 | .^c^ |  |  |  |  |
|  |  | WS-5 | WS-35 | .^c^ |  |  |  |  |
|  |  |  | WS-10 | .^b,c^ |  |  |  |  |
|  |  |  | WS-2.5 | .^c^ |  |  |  |  |
|  |  | WS-2.5 | WS-35 | 1.212^*^ | 0.114 | 0.000 | 1.024 | 1.401 |
|  |  |  | WS-10 | .^b^ |  |  |  |  |
|  |  |  | WS-5 | .^b^ |  |  |  |  |
|  | Total Withanolides | WS-35 | WS-10 | -1.057^*^ | 0.116 | 0.000 | -1.248 | -0.865 |
|  |  |  | WS-5 | -1.265^*^ | 0.114 | 0.000 | -1.453 | -1.076 |
|  |  |  | WS-2.5 | -1.619^*^ | 0.114 | 0.000 | -1.807 | -1.431 |
|  |  | WS-10 | WS-35 | 1.057^*^ | 0.116 | 0.000 | 0.865 | 1.248 |
|  |  |  | WS-5 | -.208 | 0.118 | 0.079 | -0.403 | -0.013 |
|  |  |  | WS-2.5 | -.563^*^ | 0.118 | 0.000 | -0.757 | -0.368 |
|  |  | WS-5 | WS-35 | 1.265^*^ | 0.114 | 0.000 | 1.076 | 1.453 |
|  |  |  | WS-10 | .208 | 0.118 | 0.079 | 0.013 | 0.403 |
|  |  |  | WS-2.5 | -.354^*^ | 0.116 | 0.003 | -0.546 | -0.163 |
|  |  | WS-2.5 | WS-35 | 1.619^*^ | 0.114 | 0.000 | 1.431 | 1.807 |
|  |  |  | WS-10 | .563^*^ | 0.118 | 0.000 | 0.368 | 0.757 |
|  |  |  | WS-5 | .354^*^ | 0.116 | 0.003 | 0.163 | 0.546 |
| t-half | Withanolide A | WS-35 | WS-10 | .866^*^ | 0.116 | 0.000 | 0.674 | 1.058 |
|  |  |  | WS-5 | .844^*^ | 0.114 | 0.000 | 0.656 | 1.033 |
|  |  |  | WS-2.5 | .786^*^ | 0.114 | 0.000 | 0.597 | 0.974 |
|  |  | WS-10 | WS-35 | -.866^*^ | 0.116 | 0.000 | -1.058 | -0.674 |
|  |  |  | WS-5 | -0.022 | 0.118 | 0.854 | -0.216 | 0.173 |
|  |  |  | WS-2.5 | -0.080 | 0.118 | 0.497 | -0.275 | 0.115 |
|  |  | WS-5 | WS-35 | -.844^*^ | 0.114 | 0.000 | -1.033 | -0.656 |
|  |  |  | WS-10 | 0.022 | 0.118 | 0.854 | -0.173 | 0.216 |
|  |  |  | WS-2.5 | -0.059 | 0.116 | 0.614 | -0.250 | 0.133 |
|  |  | WS-2.5 | WS-35 | -.786^*^ | 0.114 | 0.000 | -0.974 | -0.597 |
|  |  |  | WS-10 | 0.080 | 0.118 | 0.497 | -0.115 | 0.275 |
|  |  |  | WS-5 | 0.059 | 0.116 | 0.614 | -0.133 | 0.250 |
|  | Withaferin A | WS-35 | WS-10 | 1.089^*^ | 0.116 | 0.000 | 0.897 | 1.281 |
|  |  |  | WS-5 | 1.219^*^ | 0.114 | 0.000 | 1.031 | 1.407 |
|  |  |  | WS-2.5 | 1.265^*^ | 0.114 | 0.000 | 1.077 | 1.454 |
|  |  | WS-10 | WS-35 | -1.089^*^ | 0.116 | 0.000 | -1.281 | -0.897 |
|  |  |  | WS-5 | 0.130 | 0.118 | 0.273 | -0.065 | 0.324 |
|  |  |  | WS-2.5 | 0.176 | 0.118 | 0.137 | -0.019 | 0.371 |
|  |  | WS-5 | WS-35 | -1.219^*^ | 0.114 | 0.000 | -1.407 | -1.031 |
|  |  |  | WS-10 | -0.130 | 0.118 | 0.273 | -0.324 | 0.065 |
|  |  |  | WS-2.5 | 0.046 | 0.116 | 0.690 | -0.145 | 0.238 |
|  |  | WS-2.5 | WS-35 | -1.265^*^ | 0.114 | 0.000 | -1.454 | -1.077 |
|  |  |  | WS-10 | -0.176 | 0.118 | 0.137 | -0.371 | 0.019 |
|  |  |  | WS-5 | -0.046 | 0.116 | 0.690 | -0.238 | 0.145 |
|  | Withanoside IV | WS-35 | WS-10 | .^b^ |  |  |  |  |
|  |  |  | WS-5 | .^b^ |  |  |  |  |
|  |  |  | WS-2.5 | 1.212^*^ | 0.114 | 0.000 | 1.024 | 1.401 |
|  |  | WS-10 | WS-35 | .^c^ |  |  |  |  |
|  |  |  | WS-5 | .^b,c^ |  |  |  |  |
|  |  |  | WS-2.5 | .^c^ |  |  |  |  |
|  |  | WS-5 | WS-35 | .^c^ |  |  |  |  |
|  |  |  | WS-10 | .^b,c^ |  |  |  |  |
|  |  |  | WS-2.5 | .^c^ |  |  |  |  |
|  |  | WS-2.5 | WS-35 | -1.212^*^ | 0.114 | 0.000 | -1.401 | -1.024 |
|  |  |  | WS-10 | .^b^ |  |  |  |  |
|  |  |  | WS-5 | .^b^ |  |  |  |  |
|  | Total Withanolides | WS-35 | WS-10 | 1.057^*^ | 0.116 | 0.000 | 0.865 | 1.248 |
|  |  |  | WS-5 | 1.265^*^ | 0.114 | 0.000 | 1.076 | 1.453 |
|  |  |  | WS-2.5 | 1.619^*^ | 0.114 | 0.000 | 1.431 | 1.807 |
|  |  | WS-10 | WS-35 | -1.057^*^ | 0.116 | 0.000 | -1.248 | -0.865 |
|  |  |  | WS-5 | .208 | 0.118 | 0.079 | 0.013 | 0.403 |
|  |  |  | WS-2.5 | .563^*^ | 0.118 | 0.000 | 0.368 | 0.757 |
|  |  | WS-5 | WS-35 | -1.265^*^ | 0.114 | 0.000 | -1.453 | -1.076 |
|  |  |  | WS-10 | -.208 | 0.118 | 0.079 | -0.403 | -0.013 |
|  |  |  | WS-2.5 | .354^*^ | 0.116 | 0.003 | 0.163 | 0.546 |
|  |  | WS-2.5 | WS-35 | -1.619^*^ | 0.114 | 0.000 | -1.807 | -1.431 |
|  |  |  | WS-10 | -.563^*^ | 0.118 | 0.000 | -0.757 | -0.368 |
|  |  |  | WS-5 | -.354^*^ | 0.116 | 0.003 | -0.546 | -0.163 |
| Cmax | Withanolide A | WS-35 | WS-10 | .482^*^ | 0.042 | 0.000 | 0.412 | 0.551 |
|  |  |  | WS-5 | .594^*^ | 0.041 | 0.000 | 0.525 | 0.662 |
|  |  |  | WS-2.5 | .462^*^ | 0.041 | 0.000 | 0.393 | 0.530 |
|  |  | WS-10 | WS-35 | -.482^*^ | 0.042 | 0.000 | -0.551 | -0.412 |
|  |  |  | WS-5 | .112^*^ | 0.043 | 0.009 | 0.041 | 0.182 |
|  |  |  | WS-2.5 | -0.020 | 0.043 | 0.638 | -0.091 | 0.050 |
|  |  | WS-5 | WS-35 | -.594^*^ | 0.041 | 0.000 | -0.662 | -0.525 |
|  |  |  | WS-10 | -.112^*^ | 0.043 | 0.009 | -0.182 | -0.041 |
|  |  |  | WS-2.5 | -.132^*^ | 0.042 | 0.002 | -0.201 | -0.063 |
|  |  | WS-2.5 | WS-35 | -.462^*^ | 0.041 | 0.000 | -0.530 | -0.393 |
|  |  |  | WS-10 | 0.020 | 0.043 | 0.638 | -0.050 | 0.091 |
|  |  |  | WS-5 | .132^*^ | 0.042 | 0.002 | 0.063 | 0.201 |
|  | Withaferin A | WS-35 | WS-10 | 2.870^*^ | 0.042 | 0.000 | 2.801 | 2.940 |
|  |  |  | WS-5 | 2.476^*^ | 0.041 | 0.000 | 2.408 | 2.545 |
|  |  |  | WS-2.5 | 2.121^*^ | 0.041 | 0.000 | 2.053 | 2.189 |
|  |  | WS-10 | WS-35 | -2.870^*^ | 0.042 | 0.000 | -2.940 | -2.801 |
|  |  |  | WS-5 | -.394^*^ | 0.043 | 0.000 | -0.464 | -0.323 |
|  |  |  | WS-2.5 | -.749^*^ | 0.043 | 0.000 | -0.820 | -0.679 |
|  |  | WS-5 | WS-35 | -2.476^*^ | 0.041 | 0.000 | -2.545 | -2.408 |
|  |  |  | WS-10 | .394^*^ | 0.043 | 0.000 | 0.323 | 0.464 |
|  |  |  | WS-2.5 | -.356^*^ | 0.042 | 0.000 | -0.425 | -0.286 |
|  |  | WS-2.5 | WS-35 | -2.121^*^ | 0.041 | 0.000 | -2.189 | -2.053 |
|  |  |  | WS-10 | .749^*^ | 0.043 | 0.000 | 0.679 | 0.820 |
|  |  |  | WS-5 | .356^*^ | 0.042 | 0.000 | 0.286 | 0.425 |
|  | Withanoside IV | WS-35 | WS-10 | .^b^ |  |  |  |  |
|  |  |  | WS-5 | .^b^ |  |  |  |  |
|  |  |  | WS-2.5 | .997^*^ | 0.041 | 0.000 | 0.929 | 1.065 |
|  |  | WS-10 | WS-35 | .^c^ |  |  |  |  |
|  |  |  | WS-5 | .^b,c^ |  |  |  |  |
|  |  |  | WS-2.5 | .^c^ |  |  |  |  |
|  |  | WS-5 | WS-35 | .^c^ |  |  |  |  |
|  |  |  | WS-10 | .^b,c^ |  |  |  |  |
|  |  |  | WS-2.5 | .^c^ |  |  |  |  |
|  |  | WS-2.5 | WS-35 | -.997^*^ | 0.041 | 0.000 | -1.065 | -0.929 |
|  |  |  | WS-10 | .^b^ |  |  |  |  |
|  |  |  | WS-5 | .^b^ |  |  |  |  |
|  | Total Withanolides | WS-35 | WS-10 | 2.378^*^ | 0.042 | 0.000 | 2.308 | 2.447 |
|  |  |  | WS-5 | 2.256^*^ | 0.041 | 0.000 | 2.187 | 2.324 |
|  |  |  | WS-2.5 | 1.722^*^ | 0.041 | 0.000 | 1.654 | 1.790 |
|  |  | WS-10 | WS-35 | -2.378^*^ | 0.042 | 0.000 | -2.447 | -2.308 |
|  |  |  | WS-5 | -.122^*^ | 0.043 | 0.005 | -0.193 | -0.052 |
|  |  |  | WS-2.5 | -.656^*^ | 0.043 | 0.000 | -0.727 | -0.586 |
|  |  | WS-5 | WS-35 | -2.256^*^ | 0.041 | 0.000 | -2.324 | -2.187 |
|  |  |  | WS-10 | .122^*^ | 0.043 | 0.005 | 0.052 | 0.193 |
|  |  |  | WS-2.5 | -.534^*^ | 0.042 | 0.000 | -0.603 | -0.465 |
|  |  | WS-2.5 | WS-35 | -1.722^*^ | 0.041 | 0.000 | -1.790 | -1.654 |
|  |  |  | WS-10 | .656^*^ | 0.043 | 0.000 | 0.586 | 0.727 |
|  |  |  | WS-5 | .534^*^ | 0.042 | 0.000 | 0.465 | 0.603 |
| AUC0-t | Withanolide A | WS-35 | WS-10 | .935^*^ | 0.054 | 0.000 | 0.845 | 1.024 |
|  |  |  | WS-5 | 1.094^*^ | 0.053 | 0.000 | 1.007 | 1.182 |
|  |  |  | WS-2.5 | .923^*^ | 0.053 | 0.000 | 0.835 | 1.011 |
|  |  | WS-10 | WS-35 | -.935^*^ | 0.054 | 0.000 | -1.024 | -0.845 |
|  |  |  | WS-5 | .160^*^ | 0.055 | 0.004 | 0.069 | 0.251 |
|  |  |  | WS-2.5 | -0.012 | 0.055 | 0.829 | -0.103 | 0.079 |
|  |  | WS-5 | WS-35 | -1.094^*^ | 0.053 | 0.000 | -1.182 | -1.007 |
|  |  |  | WS-10 | -.160^*^ | 0.055 | 0.004 | -0.251 | -0.069 |
|  |  |  | WS-2.5 | -.172^*^ | 0.054 | 0.002 | -0.261 | -0.082 |
|  |  | WS-2.5 | WS-35 | -.923^*^ | 0.053 | 0.000 | -1.011 | -0.835 |
|  |  |  | WS-10 | 0.012 | 0.055 | 0.829 | -0.079 | 0.103 |
|  |  |  | WS-5 | .172^*^ | 0.054 | 0.002 | 0.082 | 0.261 |
|  | Withaferin A | WS-35 | WS-10 | 4.026^*^ | 0.054 | 0.000 | 3.937 | 4.116 |
|  |  |  | WS-5 | 3.779^*^ | 0.053 | 0.000 | 3.691 | 3.867 |
|  |  |  | WS-2.5 | 3.377^*^ | 0.053 | 0.000 | 3.290 | 3.465 |
|  |  | WS-10 | WS-35 | -4.026^*^ | 0.054 | 0.000 | -4.116 | -3.937 |
|  |  |  | WS-5 | -.247^*^ | 0.055 | 0.000 | -0.338 | -0.156 |
|  |  |  | WS-2.5 | -.649^*^ | 0.055 | 0.000 | -0.740 | -0.558 |
|  |  | WS-5 | WS-35 | -3.779^*^ | 0.053 | 0.000 | -3.867 | -3.691 |
|  |  |  | WS-10 | .247^*^ | 0.055 | 0.000 | 0.156 | 0.338 |
|  |  |  | WS-2.5 | -.402^*^ | 0.054 | 0.000 | -0.491 | -0.313 |
|  |  | WS-2.5 | WS-35 | -3.377^*^ | 0.053 | 0.000 | -3.465 | -3.290 |
|  |  |  | WS-10 | .649^*^ | 0.055 | 0.000 | 0.558 | 0.740 |
|  |  |  | WS-5 | .402^*^ | 0.054 | 0.000 | 0.313 | 0.491 |
|  | Withanoside IV | WS-35 | WS-10 | .^b^ |  |  |  |  |
|  |  |  | WS-5 | .^b^ |  |  |  |  |
|  |  |  | WS-2.5 | 1.662^*^ | 0.053 | 0.000 | 1.575 | 1.750 |
|  |  | WS-10 | WS-35 | .^c^ |  |  |  |  |
|  |  |  | WS-5 | .^b,c^ |  |  |  |  |
|  |  |  | WS-2.5 | .^c^ |  |  |  |  |
|  |  | WS-5 | WS-35 | .^c^ |  |  |  |  |
|  |  |  | WS-10 | .^b,c^ |  |  |  |  |
|  |  |  | WS-2.5 | .^c^ |  |  |  |  |
|  |  | WS-2.5 | WS-35 | -1.662^*^ | 0.053 | 0.000 | -1.750 | -1.575 |
|  |  |  | WS-10 | .^b^ |  |  |  |  |
|  |  |  | WS-5 | .^b^ |  |  |  |  |
|  | Total Withanolides | WS-35 | WS-10 | 3.449^*^ | 0.054 | 0.000 | 3.360 | 3.539 |
|  |  |  | WS-5 | 3.375^*^ | 0.053 | 0.000 | 3.287 | 3.463 |
|  |  |  | WS-2.5 | 2.851^*^ | 0.053 | 0.000 | 2.763 | 2.939 |
|  |  | WS-10 | WS-35 | -3.449^*^ | 0.054 | 0.000 | -3.539 | -3.360 |
|  |  |  | WS-5 | -0.074 | 0.055 | 0.177 | -0.165 | 0.016 |
|  |  |  | WS-2.5 | -.599^*^ | 0.055 | 0.000 | -0.689 | -0.508 |
|  |  | WS-5 | WS-35 | -3.375^*^ | 0.053 | 0.000 | -3.463 | -3.287 |
|  |  |  | WS-10 | 0.074 | 0.055 | 0.177 | -0.016 | 0.165 |
|  |  |  | WS-2.5 | -.524^*^ | 0.054 | 0.000 | -0.613 | -0.435 |
|  |  | WS-2.5 | WS-35 | -2.851^*^ | 0.053 | 0.000 | -2.939 | -2.763 |
|  |  |  | WS-10 | .599^*^ | 0.055 | 0.000 | 0.508 | 0.689 |
|  |  |  | WS-5 | .524^*^ | 0.054 | 0.000 | 0.435 | 0.613 |
| AUC0-inf | Withanolide A | WS-35 | WS-10 | 1.204^*^ | 0.093 | 0.000 | 1.050 | 1.358 |
|  |  |  | WS-5 | 1.308^*^ | 0.092 | 0.000 | 1.157 | 1.460 |
|  |  |  | WS-2.5 | 1.169^*^ | 0.092 | 0.000 | 1.018 | 1.321 |
|  |  | WS-10 | WS-35 | -1.204^*^ | 0.093 | 0.000 | -1.358 | -1.050 |
|  |  |  | WS-5 | 0.104 | 0.095 | 0.274 | -0.053 | 0.261 |
|  |  |  | WS-2.5 | -0.035 | 0.095 | 0.713 | -0.191 | 0.122 |
|  |  | WS-5 | WS-35 | -1.308^*^ | 0.092 | 0.000 | -1.460 | -1.157 |
|  |  |  | WS-10 | -0.104 | 0.095 | 0.274 | -0.261 | 0.053 |
|  |  |  | WS-2.5 | -0.139 | 0.093 | 0.138 | -0.293 | 0.015 |
|  |  | WS-2.5 | WS-35 | -1.169^*^ | 0.092 | 0.000 | -1.321 | -1.018 |
|  |  |  | WS-10 | 0.035 | 0.095 | 0.713 | -0.122 | 0.191 |
|  |  |  | WS-5 | 0.139 | 0.093 | 0.138 | -0.015 | 0.293 |
|  | Withaferin A | WS-35 | WS-10 | 3.869^*^ | 0.093 | 0.000 | 3.715 | 4.023 |
|  |  |  | WS-5 | 3.723^*^ | 0.092 | 0.000 | 3.572 | 3.875 |
|  |  |  | WS-2.5 | 3.346^*^ | 0.092 | 0.000 | 3.195 | 3.498 |
|  |  | WS-10 | WS-35 | -3.869^*^ | 0.093 | 0.000 | -4.023 | -3.715 |
|  |  |  | WS-5 | -0.145 | 0.095 | 0.126 | -0.302 | 0.011 |
|  |  |  | WS-2.5 | -.523^*^ | 0.095 | 0.000 | -0.679 | -0.366 |
|  |  | WS-5 | WS-35 | -3.723^*^ | 0.092 | 0.000 | -3.875 | -3.572 |
|  |  |  | WS-10 | 0.145 | 0.095 | 0.126 | -0.011 | 0.302 |
|  |  |  | WS-2.5 | -.377^*^ | 0.093 | 0.000 | -0.531 | -0.223 |
|  |  | WS-2.5 | WS-35 | -3.346^*^ | 0.092 | 0.000 | -3.498 | -3.195 |
|  |  |  | WS-10 | .523^*^ | 0.095 | 0.000 | 0.366 | 0.679 |
|  |  |  | WS-5 | .377^*^ | 0.093 | 0.000 | 0.223 | 0.531 |
|  | Withanoside IV | WS-35 | WS-10 | .^b^ |  |  |  |  |
|  |  |  | WS-5 | .^b^ |  |  |  |  |
|  |  |  | WS-2.5 | 1.987^*^ | 0.092 | 0.000 | 1.835 | 2.138 |
|  |  | WS-10 | WS-35 | .^c^ |  |  |  |  |
|  |  |  | WS-5 | .^b,c^ |  |  |  |  |
|  |  |  | WS-2.5 | .^c^ |  |  |  |  |
|  |  | WS-5 | WS-35 | .^c^ |  |  |  |  |
|  |  |  | WS-10 | .^b,c^ |  |  |  |  |
|  |  |  | WS-2.5 | .^c^ |  |  |  |  |
|  |  | WS-2.5 | WS-35 | -1.987^*^ | 0.092 | 0.000 | -2.138 | -1.835 |
|  |  |  | WS-10 | .^b^ |  |  |  |  |
|  |  |  | WS-5 | .^b^ |  |  |  |  |
|  | Total Withanolides | WS-35 | WS-10 | 3.262^*^ | 0.093 | 0.000 | 3.107 | 3.416 |
|  |  |  | WS-5 | 3.319^*^ | 0.092 | 0.000 | 3.167 | 3.470 |
|  |  |  | WS-2.5 | 2.885^*^ | 0.092 | 0.000 | 2.734 | 3.037 |
|  |  | WS-10 | WS-35 | -3.262^*^ | 0.093 | 0.000 | -3.416 | -3.107 |
|  |  |  | WS-5 | 0.057 | 0.095 | 0.549 | -0.100 | 0.214 |
|  |  |  | WS-2.5 | -.376^*^ | 0.095 | 0.000 | -0.533 | -0.220 |
|  |  | WS-5 | WS-35 | -3.319^*^ | 0.092 | 0.000 | -3.470 | -3.167 |
|  |  |  | WS-10 | -0.057 | 0.095 | 0.549 | -0.214 | 0.100 |
|  |  |  | WS-2.5 | -.433^*^ | 0.093 | 0.000 | -0.587 | -0.279 |
|  |  | WS-2.5 | WS-35 | -2.885^*^ | 0.092 | 0.000 | -3.037 | -2.734 |
|  |  |  | WS-10 | .376^*^ | 0.095 | 0.000 | 0.220 | 0.533 |
|  |  |  | WS-5 | .433^*^ | 0.093 | 0.000 | 0.279 | 0.587 |
| AUCext | Withanolide A | WS-35 | WS-10 | 1.497^*^ | 0.167 | 0.000 | 1.221 | 1.773 |
|  |  |  | WS-5 | 1.496^*^ | 0.164 | 0.000 | 1.225 | 1.767 |
|  |  |  | WS-2.5 | 1.407^*^ | 0.164 | 0.000 | 1.135 | 1.678 |
|  |  | WS-10 | WS-35 | -1.497^*^ | 0.167 | 0.000 | -1.773 | -1.221 |
|  |  |  | WS-5 | -0.001 | 0.170 | 0.997 | -0.281 | 0.280 |
|  |  |  | WS-2.5 | -0.090 | 0.170 | 0.596 | -0.370 | 0.190 |
|  |  | WS-5 | WS-35 | -1.496^*^ | 0.164 | 0.000 | -1.767 | -1.225 |
|  |  |  | WS-10 | 0.001 | 0.170 | 0.997 | -0.280 | 0.281 |
|  |  |  | WS-2.5 | -0.089 | 0.167 | 0.593 | -0.365 | 0.186 |
|  |  | WS-2.5 | WS-35 | -1.407^*^ | 0.164 | 0.000 | -1.678 | -1.135 |
|  |  |  | WS-10 | 0.090 | 0.170 | 0.596 | -0.190 | 0.370 |
|  |  |  | WS-5 | 0.089 | 0.167 | 0.593 | -0.186 | 0.365 |
|  | Withaferin A | WS-35 | WS-10 | 3.459^*^ | 0.167 | 0.000 | 3.183 | 3.735 |
|  |  |  | WS-5 | 3.557^*^ | 0.164 | 0.000 | 3.286 | 3.828 |
|  |  |  | WS-2.5 | 3.241^*^ | 0.164 | 0.000 | 2.970 | 3.512 |
|  |  | WS-10 | WS-35 | -3.459^*^ | 0.167 | 0.000 | -3.735 | -3.183 |
|  |  |  | WS-5 | 0.098 | 0.170 | 0.564 | -0.182 | 0.379 |
|  |  |  | WS-2.5 | -0.218 | 0.170 | 0.201 | -0.498 | 0.063 |
|  |  | WS-5 | WS-35 | -3.557^*^ | 0.164 | 0.000 | -3.828 | -3.286 |
|  |  |  | WS-10 | -0.098 | 0.170 | 0.564 | -0.379 | 0.182 |
|  |  |  | WS-2.5 | -.316 | 0.167 | 0.060 | -0.591 | -0.040 |
|  |  | WS-2.5 | WS-35 | -3.241^*^ | 0.164 | 0.000 | -3.512 | -2.970 |
|  |  |  | WS-10 | 0.218 | 0.170 | 0.201 | -0.063 | 0.498 |
|  |  |  | WS-5 | .316 | 0.167 | 0.060 | 0.040 | 0.591 |
|  | Withanoside IV | WS-35 | WS-10 | .^b^ |  |  |  |  |
|  |  |  | WS-5 | .^b^ |  |  |  |  |
|  |  |  | WS-2.5 | 2.311^*^ | 0.164 | 0.000 | 2.040 | 2.582 |
|  |  | WS-10 | WS-35 | .^c^ |  |  |  |  |
|  |  |  | WS-5 | .^b,c^ |  |  |  |  |
|  |  |  | WS-2.5 | .^c^ |  |  |  |  |
|  |  | WS-5 | WS-35 | .^c^ |  |  |  |  |
|  |  |  | WS-10 | .^b,c^ |  |  |  |  |
|  |  |  | WS-2.5 | .^c^ |  |  |  |  |
|  |  | WS-2.5 | WS-35 | -2.311^*^ | 0.164 | 0.000 | -2.582 | -2.040 |
|  |  |  | WS-10 | .^b^ |  |  |  |  |
|  |  |  | WS-5 | .^b^ |  |  |  |  |
|  | Total Withanolides | WS-35 | WS-10 | 2.689^*^ | 0.167 | 0.000 | 2.413 | 2.965 |
|  |  |  | WS-5 | 3.206^*^ | 0.164 | 0.000 | 2.935 | 3.477 |
|  |  |  | WS-2.5 | 3.026^*^ | 0.164 | 0.000 | 2.755 | 3.297 |
|  |  | WS-10 | WS-35 | -2.689^*^ | 0.167 | 0.000 | -2.965 | -2.413 |
|  |  |  | WS-5 | .517^*^ | 0.170 | 0.003 | 0.236 | 0.797 |
|  |  |  | WS-2.5 | .337^*^ | 0.170 | 0.049 | 0.056 | 0.617 |
|  |  | WS-5 | WS-35 | -3.206^*^ | 0.164 | 0.000 | -3.477 | -2.935 |
|  |  |  | WS-10 | -.517^*^ | 0.170 | 0.003 | -0.797 | -0.236 |
|  |  |  | WS-2.5 | -0.180 | 0.167 | 0.282 | -0.455 | 0.096 |
|  |  | WS-2.5 | WS-35 | -3.026^*^ | 0.164 | 0.000 | -3.297 | -2.755 |
|  |  |  | WS-10 | -.337^*^ | 0.170 | 0.049 | -0.617 | -0.056 |
|  |  |  | WS-5 | 0.180 | 0.167 | 0.282 | -0.096 | 0.455 |
| MRT | Withanolide A | WS-35 | WS-10 | .787^*^ | 0.098 | 0.000 | 0.626 | 0.949 |
|  |  |  | WS-5 | .785^*^ | 0.096 | 0.000 | 0.626 | 0.943 |
|  |  |  | WS-2.5 | .744^*^ | 0.096 | 0.000 | 0.585 | 0.902 |
|  |  | WS-10 | WS-35 | -.787^*^ | 0.098 | 0.000 | -0.949 | -0.626 |
|  |  |  | WS-5 | -0.003 | 0.099 | 0.979 | -0.167 | 0.161 |
|  |  |  | WS-2.5 | -0.043 | 0.099 | 0.662 | -0.207 | 0.121 |
|  |  | WS-5 | WS-35 | -.785^*^ | 0.096 | 0.000 | -0.943 | -0.626 |
|  |  |  | WS-10 | 0.003 | 0.099 | 0.979 | -0.161 | 0.167 |
|  |  |  | WS-2.5 | -0.041 | 0.097 | 0.677 | -0.202 | 0.120 |
|  |  | WS-2.5 | WS-35 | -.744^*^ | 0.096 | 0.000 | -0.902 | -0.585 |
|  |  |  | WS-10 | 0.043 | 0.099 | 0.662 | -0.121 | 0.207 |
|  |  |  | WS-5 | 0.041 | 0.097 | 0.677 | -0.120 | 0.202 |
|  | Withaferin A | WS-35 | WS-10 | 1.060^*^ | 0.098 | 0.000 | 0.899 | 1.222 |
|  |  |  | WS-5 | 1.251^*^ | 0.096 | 0.000 | 1.092 | 1.409 |
|  |  |  | WS-2.5 | 1.290^*^ | 0.096 | 0.000 | 1.132 | 1.449 |
|  |  | WS-10 | WS-35 | -1.060^*^ | 0.098 | 0.000 | -1.222 | -0.899 |
|  |  |  | WS-5 | .191 | 0.099 | 0.056 | 0.027 | 0.354 |
|  |  |  | WS-2.5 | .230^*^ | 0.099 | 0.022 | 0.066 | 0.394 |
|  |  | WS-5 | WS-35 | -1.251^*^ | 0.096 | 0.000 | -1.409 | -1.092 |
|  |  |  | WS-10 | -.191 | 0.099 | 0.056 | -0.354 | -0.027 |
|  |  |  | WS-2.5 | 0.039 | 0.097 | 0.688 | -0.122 | 0.200 |
|  |  | WS-2.5 | WS-35 | -1.290^*^ | 0.096 | 0.000 | -1.449 | -1.132 |
|  |  |  | WS-10 | -.230^*^ | 0.099 | 0.022 | -0.394 | -0.066 |
|  |  |  | WS-5 | -0.039 | 0.097 | 0.688 | -0.200 | 0.122 |
|  | Withanoside IV | WS-35 | WS-10 | .^b^ |  |  |  |  |
|  |  |  | WS-5 | .^b^ |  |  |  |  |
|  |  |  | WS-2.5 | 1.044^*^ | 0.096 | 0.000 | 0.885 | 1.202 |
|  |  | WS-10 | WS-35 | .^c^ |  |  |  |  |
|  |  |  | WS-5 | .^b,c^ |  |  |  |  |
|  |  |  | WS-2.5 | .^c^ |  |  |  |  |
|  |  | WS-5 | WS-35 | .^c^ |  |  |  |  |
|  |  |  | WS-10 | .^b,c^ |  |  |  |  |
|  |  |  | WS-2.5 | .^c^ |  |  |  |  |
|  |  | WS-2.5 | WS-35 | -1.044^*^ | 0.096 | 0.000 | -1.202 | -0.885 |
|  |  |  | WS-10 | .^b^ |  |  |  |  |
|  |  |  | WS-5 | .^b^ |  |  |  |  |
|  | Total Withanolides | WS-35 | WS-10 | .964^*^ | 0.098 | 0.000 | 0.803 | 1.126 |
|  |  |  | WS-5 | 1.201^*^ | 0.096 | 0.000 | 1.043 | 1.360 |
|  |  |  | WS-2.5 | 1.398^*^ | 0.096 | 0.000 | 1.239 | 1.556 |
|  |  | WS-10 | WS-35 | -.964^*^ | 0.098 | 0.000 | -1.126 | -0.803 |
|  |  |  | WS-5 | .237^*^ | 0.099 | 0.018 | 0.073 | 0.401 |
|  |  |  | WS-2.5 | .434^*^ | 0.099 | 0.000 | 0.270 | 0.597 |
|  |  | WS-5 | WS-35 | -1.201^*^ | 0.096 | 0.000 | -1.360 | -1.043 |
|  |  |  | WS-10 | -.237^*^ | 0.099 | 0.018 | -0.401 | -0.073 |
|  |  |  | WS-2.5 | .197^*^ | 0.097 | 0.045 | 0.036 | 0.358 |
|  |  | WS-2.5 | WS-35 | -1.398^*^ | 0.096 | 0.000 | -1.556 | -1.239 |
|  |  |  | WS-10 | -.434^*^ | 0.099 | 0.000 | -0.597 | -0.270 |
|  |  |  | WS-5 | -.197^*^ | 0.097 | 0.045 | -0.358 | -0.036 |
| Vz | Withanolide A | WS-35 | WS-10 | -.338^*^ | 0.071 | 0.000 | -0.456 | -0.221 |
|  |  |  | WS-5 | -.464^*^ | 0.070 | 0.000 | -0.579 | -0.349 |
|  |  |  | WS-2.5 | -.384^*^ | 0.070 | 0.000 | -0.499 | -0.268 |
|  |  | WS-10 | WS-35 | .338^*^ | 0.071 | 0.000 | 0.221 | 0.456 |
|  |  |  | WS-5 | -.126 | 0.072 | 0.083 | -0.245 | -0.007 |
|  |  |  | WS-2.5 | -0.045 | 0.072 | 0.530 | -0.164 | 0.074 |
|  |  | WS-5 | WS-35 | .464^*^ | 0.070 | 0.000 | 0.349 | 0.579 |
|  |  |  | WS-10 | .126 | 0.072 | 0.083 | 0.007 | 0.245 |
|  |  |  | WS-2.5 | 0.080 | 0.071 | 0.259 | -0.037 | 0.197 |
|  |  | WS-2.5 | WS-35 | .384^*^ | 0.070 | 0.000 | 0.268 | 0.499 |
|  |  |  | WS-10 | 0.045 | 0.072 | 0.530 | -0.074 | 0.164 |
|  |  |  | WS-5 | -0.080 | 0.071 | 0.259 | -0.197 | 0.037 |
|  | Withaferin A | WS-35 | WS-10 | -2.779^*^ | 0.071 | 0.000 | -2.897 | -2.662 |
|  |  |  | WS-5 | -2.504^*^ | 0.070 | 0.000 | -2.620 | -2.389 |
|  |  |  | WS-2.5 | -2.081^*^ | 0.070 | 0.000 | -2.196 | -1.966 |
|  |  | WS-10 | WS-35 | 2.779^*^ | 0.071 | 0.000 | 2.662 | 2.897 |
|  |  |  | WS-5 | .275^*^ | 0.072 | 0.000 | 0.156 | 0.394 |
|  |  |  | WS-2.5 | .699^*^ | 0.072 | 0.000 | 0.579 | 0.818 |
|  |  | WS-5 | WS-35 | 2.504^*^ | 0.070 | 0.000 | 2.389 | 2.620 |
|  |  |  | WS-10 | -.275^*^ | 0.072 | 0.000 | -0.394 | -0.156 |
|  |  |  | WS-2.5 | .423^*^ | 0.071 | 0.000 | 0.306 | 0.540 |
|  |  | WS-2.5 | WS-35 | 2.081^*^ | 0.070 | 0.000 | 1.966 | 2.196 |
|  |  |  | WS-10 | -.699^*^ | 0.072 | 0.000 | -0.818 | -0.579 |
|  |  |  | WS-5 | -.423^*^ | 0.071 | 0.000 | -0.540 | -0.306 |
|  | Withanoside IV | WS-35 | WS-10 | .^b^ |  |  |  |  |
|  |  |  | WS-5 | .^b^ |  |  |  |  |
|  |  |  | WS-2.5 | -.775^*^ | 0.070 | 0.000 | -0.890 | -0.660 |
|  |  | WS-10 | WS-35 | .^c^ |  |  |  |  |
|  |  |  | WS-5 | .^b,c^ |  |  |  |  |
|  |  |  | WS-2.5 | .^c^ |  |  |  |  |
|  |  | WS-5 | WS-35 | .^c^ |  |  |  |  |
|  |  |  | WS-10 | .^b,c^ |  |  |  |  |
|  |  |  | WS-2.5 | .^c^ |  |  |  |  |
|  |  | WS-2.5 | WS-35 | .775^*^ | 0.070 | 0.000 | 0.660 | 0.890 |
|  |  |  | WS-10 | .^b^ |  |  |  |  |
|  |  |  | WS-5 | .^b^ |  |  |  |  |
|  | Total Withanolides | WS-35 | WS-10 | -2.205^*^ | 0.071 | 0.000 | -2.322 | -2.088 |
|  |  |  | WS-5 | -2.054^*^ | 0.070 | 0.000 | -2.169 | -1.939 |
|  |  |  | WS-2.5 | -1.266^*^ | 0.070 | 0.000 | -1.381 | -1.151 |
|  |  | WS-10 | WS-35 | 2.205^*^ | 0.071 | 0.000 | 2.088 | 2.322 |
|  |  |  | WS-5 | .151^*^ | 0.072 | 0.037 | 0.032 | 0.270 |
|  |  |  | WS-2.5 | .939^*^ | 0.072 | 0.000 | 0.820 | 1.058 |
|  |  | WS-5 | WS-35 | 2.054^*^ | 0.070 | 0.000 | 1.939 | 2.169 |
|  |  |  | WS-10 | -.151^*^ | 0.072 | 0.037 | -0.270 | -0.032 |
|  |  |  | WS-2.5 | .788^*^ | 0.071 | 0.000 | 0.671 | 0.905 |
|  |  | WS-2.5 | WS-35 | 1.266^*^ | 0.070 | 0.000 | 1.151 | 1.381 |
|  |  |  | WS-10 | -.939^*^ | 0.072 | 0.000 | -1.058 | -0.820 |
|  |  |  | WS-5 | -.788^*^ | 0.071 | 0.000 | -0.905 | -0.671 |
| Cl | Withanolide A | WS-35 | WS-10 | -1.204^*^ | 0.093 | 0.000 | -1.358 | -1.050 |
|  |  |  | WS-5 | -1.308^*^ | 0.092 | 0.000 | -1.460 | -1.157 |
|  |  |  | WS-2.5 | -1.169^*^ | 0.092 | 0.000 | -1.321 | -1.018 |
|  |  | WS-10 | WS-35 | 1.204^*^ | 0.093 | 0.000 | 1.050 | 1.358 |
|  |  |  | WS-5 | -0.104 | 0.095 | 0.274 | -0.261 | 0.053 |
|  |  |  | WS-2.5 | 0.035 | 0.095 | 0.713 | -0.122 | 0.191 |
|  |  | WS-5 | WS-35 | 1.308^*^ | 0.092 | 0.000 | 1.157 | 1.460 |
|  |  |  | WS-10 | 0.104 | 0.095 | 0.274 | -0.053 | 0.261 |
|  |  |  | WS-2.5 | 0.139 | 0.093 | 0.138 | -0.015 | 0.293 |
|  |  | WS-2.5 | WS-35 | 1.169^*^ | 0.092 | 0.000 | 1.018 | 1.321 |
|  |  |  | WS-10 | -0.035 | 0.095 | 0.713 | -0.191 | 0.122 |
|  |  |  | WS-5 | -0.139 | 0.093 | 0.138 | -0.293 | 0.015 |
|  | Withaferin A | WS-35 | WS-10 | -3.869^*^ | 0.093 | 0.000 | -4.023 | -3.715 |
|  |  |  | WS-5 | -3.723^*^ | 0.092 | 0.000 | -3.875 | -3.572 |
|  |  |  | WS-2.5 | -3.346^*^ | 0.092 | 0.000 | -3.498 | -3.195 |
|  |  | WS-10 | WS-35 | 3.869^*^ | 0.093 | 0.000 | 3.715 | 4.023 |
|  |  |  | WS-5 | 0.145 | 0.095 | 0.126 | -0.011 | 0.302 |
|  |  |  | WS-2.5 | .523^*^ | 0.095 | 0.000 | 0.366 | 0.679 |
|  |  | WS-5 | WS-35 | 3.723^*^ | 0.092 | 0.000 | 3.572 | 3.875 |
|  |  |  | WS-10 | -0.145 | 0.095 | 0.126 | -0.302 | 0.011 |
|  |  |  | WS-2.5 | .377^*^ | 0.093 | 0.000 | 0.223 | 0.531 |
|  |  | WS-2.5 | WS-35 | 3.346^*^ | 0.092 | 0.000 | 3.195 | 3.498 |
|  |  |  | WS-10 | -.523^*^ | 0.095 | 0.000 | -0.679 | -0.366 |
|  |  |  | WS-5 | -.377^*^ | 0.093 | 0.000 | -0.531 | -0.223 |
|  | Withanoside IV | WS-35 | WS-10 | .^b^ |  |  |  |  |
|  |  |  | WS-5 | .^b^ |  |  |  |  |
|  |  |  | WS-2.5 | -1.987^*^ | 0.092 | 0.000 | -2.138 | -1.835 |
|  |  | WS-10 | WS-35 | .^c^ |  |  |  |  |
|  |  |  | WS-5 | .^b,c^ |  |  |  |  |
|  |  |  | WS-2.5 | .^c^ |  |  |  |  |
|  |  | WS-5 | WS-35 | .^c^ |  |  |  |  |
|  |  |  | WS-10 | .^b,c^ |  |  |  |  |
|  |  |  | WS-2.5 | .^c^ |  |  |  |  |
|  |  | WS-2.5 | WS-35 | 1.987^*^ | 0.092 | 0.000 | 1.835 | 2.138 |
|  |  |  | WS-10 | .^b^ |  |  |  |  |
|  |  |  | WS-5 | .^b^ |  |  |  |  |
|  | Total Withanolides | WS-35 | WS-10 | -3.262^*^ | 0.093 | 0.000 | -3.416 | -3.107 |
|  |  |  | WS-5 | -3.319^*^ | 0.092 | 0.000 | -3.470 | -3.167 |
|  |  |  | WS-2.5 | -2.885^*^ | 0.092 | 0.000 | -3.037 | -2.734 |
|  |  | WS-10 | WS-35 | 3.262^*^ | 0.093 | 0.000 | 3.107 | 3.416 |
|  |  |  | WS-5 | -0.057 | 0.095 | 0.549 | -0.214 | 0.100 |
|  |  |  | WS-2.5 | .376^*^ | 0.095 | 0.000 | 0.220 | 0.533 |
|  |  | WS-5 | WS-35 | 3.319^*^ | 0.092 | 0.000 | 3.167 | 3.470 |
|  |  |  | WS-10 | 0.057 | 0.095 | 0.549 | -0.100 | 0.214 |
|  |  |  | WS-2.5 | .433^*^ | 0.093 | 0.000 | 0.279 | 0.587 |
|  |  | WS-2.5 | WS-35 | 2.885^*^ | 0.092 | 0.000 | 2.734 | 3.037 |
|  |  |  | WS-10 | -.376^*^ | 0.095 | 0.000 | -0.533 | -0.220 |
|  |  |  | WS-5 | -.433^*^ | 0.093 | 0.000 | -0.587 | -0.279 |
| Ke | Withanolide A | WS-35 | WS-10 | -.866^*^ | 0.116 | 0.000 | -1.058 | -0.674 |
|  |  |  | WS-5 | -.844^*^ | 0.114 | 0.000 | -1.033 | -0.656 |
|  |  |  | WS-2.5 | -.786^*^ | 0.114 | 0.000 | -0.974 | -0.597 |
|  |  | WS-10 | WS-35 | .866^*^ | 0.116 | 0.000 | 0.674 | 1.058 |
|  |  |  | WS-5 | 0.022 | 0.118 | 0.854 | -0.173 | 0.216 |
|  |  |  | WS-2.5 | 0.080 | 0.118 | 0.497 | -0.115 | 0.275 |
|  |  | WS-5 | WS-35 | .844^*^ | 0.114 | 0.000 | 0.656 | 1.033 |
|  |  |  | WS-10 | -0.022 | 0.118 | 0.854 | -0.216 | 0.173 |
|  |  |  | WS-2.5 | 0.059 | 0.116 | 0.614 | -0.133 | 0.250 |
|  |  | WS-2.5 | WS-35 | .786^*^ | 0.114 | 0.000 | 0.597 | 0.974 |
|  |  |  | WS-10 | -0.080 | 0.118 | 0.497 | -0.275 | 0.115 |
|  |  |  | WS-5 | -0.059 | 0.116 | 0.614 | -0.250 | 0.133 |
|  | Withaferin A | WS-35 | WS-10 | -1.089^*^ | 0.116 | 0.000 | -1.281 | -0.897 |
|  |  |  | WS-5 | -1.219^*^ | 0.114 | 0.000 | -1.407 | -1.031 |
|  |  |  | WS-2.5 | -1.265^*^ | 0.114 | 0.000 | -1.454 | -1.077 |
|  |  | WS-10 | WS-35 | 1.089^*^ | 0.116 | 0.000 | 0.897 | 1.281 |
|  |  |  | WS-5 | -0.130 | 0.118 | 0.273 | -0.324 | 0.065 |
|  |  |  | WS-2.5 | -0.176 | 0.118 | 0.137 | -0.371 | 0.019 |
|  |  | WS-5 | WS-35 | 1.219^*^ | 0.114 | 0.000 | 1.031 | 1.407 |
|  |  |  | WS-10 | 0.130 | 0.118 | 0.273 | -0.065 | 0.324 |
|  |  |  | WS-2.5 | -0.046 | 0.116 | 0.690 | -0.238 | 0.145 |
|  |  | WS-2.5 | WS-35 | 1.265^*^ | 0.114 | 0.000 | 1.077 | 1.454 |
|  |  |  | WS-10 | 0.176 | 0.118 | 0.137 | -0.019 | 0.371 |
|  |  |  | WS-5 | 0.046 | 0.116 | 0.690 | -0.145 | 0.238 |
|  | Withanoside IV | WS-35 | WS-10 | .^b^ |  |  |  |  |
|  |  |  | WS-5 | .^b^ |  |  |  |  |
|  |  |  | WS-2.5 | -1.212^*^ | 0.114 | 0.000 | -1.401 | -1.024 |
|  |  | WS-10 | WS-35 | .^c^ |  |  |  |  |
|  |  |  | WS-5 | .^b,c^ |  |  |  |  |
|  |  |  | WS-2.5 | .^c^ |  |  |  |  |
|  |  | WS-5 | WS-35 | .^c^ |  |  |  |  |
|  |  |  | WS-10 | .^b,c^ |  |  |  |  |
|  |  |  | WS-2.5 | .^c^ |  |  |  |  |
|  |  | WS-2.5 | WS-35 | 1.212^*^ | 0.114 | 0.000 | 1.024 | 1.401 |
|  |  |  | WS-10 | .^b^ |  |  |  |  |
|  |  |  | WS-5 | .^b^ |  |  |  |  |
|  | Total Withanolides | WS-35 | WS-10 | -1.057^*^ | 0.116 | 0.000 | -1.248 | -0.865 |
|  |  |  | WS-5 | -1.265^*^ | 0.114 | 0.000 | -1.453 | -1.076 |
|  |  |  | WS-2.5 | -1.619^*^ | 0.114 | 0.000 | -1.807 | -1.431 |
|  |  | WS-10 | WS-35 | 1.057^*^ | 0.116 | 0.000 | 0.865 | 1.248 |
|  |  |  | WS-5 | -.208 | 0.118 | 0.079 | -0.403 | -0.013 |
|  |  |  | WS-2.5 | -.563^*^ | 0.118 | 0.000 | -0.757 | -0.368 |
|  |  | WS-5 | WS-35 | 1.265^*^ | 0.114 | 0.000 | 1.076 | 1.453 |
|  |  |  | WS-10 | .208 | 0.118 | 0.079 | 0.013 | 0.403 |
|  |  |  | WS-2.5 | -.354^*^ | 0.116 | 0.003 | -0.546 | -0.163 |
|  |  | WS-2.5 | WS-35 | 1.619^*^ | 0.114 | 0.000 | 1.431 | 1.807 |
|  |  |  | WS-10 | .563^*^ | 0.118 | 0.000 | 0.368 | 0.757 |
|  |  |  | WS-5 | .354^*^ | 0.116 | 0.003 | 0.163 | 0.546 |
| Based on estimated marginal means | | | | | | | | |
| *. The mean difference is significant at the .05 level. | | | | | | | | |
| b. The level combination of factors in WS-10 is not observed. | | | | | | | | |
| c. The level combination of factors in WS-5 is not observed. | | | | | | | | |
| d. Adjustment for multiple comparisons: Least Significant Difference (equivalent to no adjustments). | | | | | | | | |

| **Comparison of Tmax** | | | | | | |
| --- | --- | --- | --- | --- | --- | --- |
| **Parameter** | **Groups** | **Count** | **Mean** | **Median** | **W*** | **P-Value** |
| Withanolide A | WS-35 | 16 | 1.813 | 2 |  |  |
|  | to WS-10 | 14 | 2.036 | 2 | 3.753 | 0.040 |
|  | to WS-5 | 15 | 2.200 | 2 | 4.464 | 0.009 |
|  | to WS-2.5 | 15 | 2.200 | 2 | 4.464 | 0.009 |
|  |  |  |  |  |  |  |
|  | WS-10 | 14 | 2.036 | 2 |  |  |
|  | to WS-35 | 16 | 1.813 | 2 | 3.753 | 0.040 |
|  | to WS-5 | 15 | 2.200 | 2 | 2.676 | 0.231 |
|  | to WS-2.5 | 15 | 2.200 | 2 | 2.676 | 0.231 |
|  |  |  |  |  |  |  |
|  | WS-5 | 15 | 2.200 | 2 |  |  |
|  | to WS-35 | 16 | 1.813 | 2 | 4.464 | 0.009 |
|  | to WS-10 | 14 | 2.036 | 2 | 2.676 | 0.231 |
|  | to WS-2.5 | 15 | 2.200 | 2 | 0.000 | 1.000 |
|  |  |  |  |  |  |  |
|  | WS-2.5 | 15 | 2.200 | 2 |  |  |
|  | to WS-35 | 16 | 1.813 | 2 | 4.464 | 0.009 |
|  | to WS-10 | 14 | 2.036 | 2 | 2.676 | 0.231 |
|  | to WS-5 | 15 | 2.200 | 2 | 0.000 | 1.000 |
|  |  |  |  |  |  |  |
| Withaferin A | WS-35 | 16 | 2.219 | 2.25 |  |  |
|  | to WS-10 | 14 | 1.964 | 2 | 3.159 | 0.114 |
|  | to WS-5 | 15 | 1.700 | 1.5 | 4.809 | 0.004 |
|  | to WS-2.5 | 15 | 1.500 | 1.5 | 6.493 | 0.000 |
|  |  |  |  |  |  |  |
|  | WS-10 | 14 | 1.964 | 2 |  |  |
|  | to WS-35 | 16 | 2.219 | 2.25 | 3.159 | 0.114 |
|  | to WS-5 | 15 | 1.700 | 1.5 | 4.158 | 0.017 |
|  | to WS-2.5 | 15 | 1.500 | 1.5 | 6.982 | 0.000 |
|  |  |  |  |  |  |  |
|  | WS-5 | 15 | 1.700 | 1.5 |  |  |
|  | to WS-35 | 16 | 2.219 | 2.25 | 4.809 | 0.004 |
|  | to WS-10 | 14 | 1.964 | 2 | 4.158 | 0.017 |
|  | to WS-2.5 | 15 | 1.500 | 1.5 | 3.808 | 0.036 |
|  |  |  |  |  |  |  |
|  | WS-2.5 | 15 | 1.500 | 1.5 |  |  |
|  | to WS-35 | 16 | 2.219 | 2.25 | 6.493 | 0.000 |
|  | to WS-10 | 14 | 1.964 | 2 | 6.982 | 0.000 |
|  | to WS-5 | 15 | 1.700 | 1.5 | 3.808 | 0.036 |
|  |  |  |  |  |  |  |
| Withanoside IV | WS-35 | 16 | 1.750 | 1.75 |  |  |
|  | to WS-2.5 | 15 | 1.567 | 1.5 | 3.391 | 0.016 |
|  |  |  |  |  |  |  |
|  | WS-2.5 | 15 | 1.567 | 1.5 |  |  |
|  | to WS-35 | 16 | 1.750 | 1.75 | 3.391 | 0.016 |
|  |  |  |  |  |  |  |
| Total Withanolides | WS-35 | 16 | 2.219 | 2.25 |  |  |
|  | to WS-10 | 14 | 2.036 | 2 | 2.447 | 0.308 |
|  | to WS-5 | 15 | 1.733 | 1.5 | 4.583 | 0.007 |
|  | to WS-2.5 | 15 | 1.500 | 1.5 | 6.493 | 0.000 |
|  |  |  |  |  |  |  |
|  | WS-10 | 14 | 2.036 | 2 |  |  |
|  | to WS-35 | 16 | 2.219 | 2.25 | 2.447 | 0.308 |
|  | to WS-5 | 15 | 1.733 | 1.5 | 4.549 | 0.007 |
|  | to WS-2.5 | 15 | 1.500 | 1.5 | 7.374 | 0.000 |
|  |  |  |  |  |  |  |
|  | WS-5 | 15 | 1.733 | 1.5 |  |  |
|  | to WS-35 | 16 | 2.219 | 2.25 | 4.583 | 0.007 |
|  | to WS-10 | 14 | 2.036 | 2 | 4.549 | 0.007 |
|  | to WS-2.5 | 15 | 1.500 | 1.5 | 4.201 | 0.016 |
|  |  |  |  |  |  |  |
|  | WS-2.5 | 15 | 1.500 | 1.5 |  |  |
|  | to WS-35 | 16 | 2.219 | 2.25 | 6.493 | 0.000 |
|  | to WS-10 | 14 | 2.036 | 2 | 7.374 | 0.000 |
|  | to WS-5 | 15 | 1.733 | 1.5 | 4.201 | 0.016 |
| * Dwass-Steel-Critchlow-Fligner Multiple Comparison test | | | | | | |
